# *OsJAZ11* regulates phosphate starvation responses in rice

**DOI:** 10.1007/s00425-021-03657-6

**Published:** 2021-06-18

**Authors:** Bipin K. Pandey, Lokesh Verma, Ankita Prusty, Ajit Pal Singh, Malcolm J. Bennett, Akhilesh K. Tyagi, Jitender Giri, Poonam Mehra

**Affiliations:** 1 National Institute of Plant Genome Research, New Delhi, 110067 India; 2 Department of Plant Molecular Biology, University of Delhi South Campus, New Delhi, 110021 India; 3 Plant and Crop Sciences, School of Biosciences, University of Nottingham, Nottingham, LE12 5RD UK

**Keywords:** Jasmonic acid, JASMONATE ZIM-DOMAIN, OsSPX1, Phosphate deficiency, Root length

## Abstract

****Main conclusion**:**

***OsJAZ11* regulates phosphate homeostasis by suppressing jasmonic acid signaling and biosynthesis in rice roots.**

**Abstract:**

Jasmonic Acid (JA) is a key plant signaling molecule which negatively regulates growth processes including root elongation. JAZ (JASMONATE ZIM-DOMAIN) proteins function as transcriptional repressors of JA signaling. Therefore, targeting JA signaling by deploying JAZ repressors may enhance root length in crops. In this study, we overexpressed JAZ repressor *OsJAZ11* in rice to alleviate the root growth inhibitory action of JA. *OsJAZ11* is a low phosphate (Pi) responsive gene which is transcriptionally regulated by OsPHR2. We report that *OsJAZ11* overexpression promoted primary and seminal root elongation which enhanced Pi foraging. Expression studies revealed that overexpression of *OsJAZ11* also reduced Pi starvation response (PSR) under Pi limiting conditions. Moreover, *OsJAZ11* overexpression also suppressed JA signaling and biosynthesis as compared to wild type (WT). We further demonstrated that the C-terminal region of OsJAZ11 was crucial for stimulating root elongation in overexpression lines. Rice transgenics overexpressing truncated *OsJAZ11ΔC* transgene (i.e., missing C-terminal region) exhibited reduced root length and Pi uptake. Interestingly, OsJAZ11 also regulates Pi homeostasis via physical interaction with a key Pi sensing protein, OsSPX1. Our study highlights the functional connections between JA and Pi signaling and reveals JAZ repressors as a promising candidate for improving low Pi tolerance of elite rice genotypes.

**Supplementary Information:**

The online version contains supplementary material available at 10.1007/s00425-021-03657-6.

## Introduction

Food security is a pressing global issue. Given the impact of climate change, feeding 9.8 billion people in 2050 poses a formidable challenge. Widespread use of chemical fertilizers has helped to deliver the “green revolution” since the 1960s. However, yields of major crops have recently plateaued and yield potential is not increasing simply through additional fertilizer application. Engineering crop plants with improved nutrient acquisition efficiency (NAE) and nutrient use efficiency (NUE) appears a promising approach to address increasing global food demands.

Phosphate (Pi) deficiency is a major problem limiting crop production worldwide impacting ~ 5.7 billion hectares of land globally (Yamaji et al. 2017). Due to its high reactivity, 80–90% of applied Pi fertilizers are rapidly fixed in soil and becomes unavailable for plant use (Gerke et al. 1994). This extensive fixing of Pi leaves farmers with no choice other than applying excessive Pi fertilizers which further aggravates demand of Pi fertilizers. Unfortunately, rock phosphates, an exclusive source of Pi fertilizers, are non-renewable and finite in nature. Therefore, indiscriminate use of Pi fertilizers threatens global food security (Cordell et al. 2009). Excess use of Pi fertilizers also pollutes lakes and other water bodies (Yamaji et al. 2017). Therefore, sustainable solutions are urgently required to alleviate Pi deficiency as well as environmental degradation. Rice, a staple food crop for more than 50% of the global population, is also one of the highest consumers of Pi fertilizers (Zhang et al. 2020). High yielding rice genotypes are ‘nutrient-exhaustive’ and demand high applications of Pi fertilizers (Mehra et al. 2016). Therefore, improving modern high yielding cultivars is necessary to boost agricultural output without additional input.

Phosphorus represents a particularly challenging nutrient owing to its low soil availability and high immobility (Lynch 2019). To enhance Pi uptake and soil foraging, plant roots have been described to exhibit a suite of adaptive responses (Pandey et al. 2013). In rice, these adaptive responses include increased primary root elongation, shallow root growth, enhanced root hair length/density and aerenchyma formation (Shimizu et al. 2004; Nestler and Wissuwa 2016; Huang et al. 2018; Pujol and Wissuwa 2018). Many of these low Pi root adaptive traits have been described to be regulated by hormones such as auxin and ethylene in rice (Giri et al. 2018; Huang et al. 2018; Lee et al. 2019). Root length is a major root trait which influences almost every other aspect of root architecture such as amplitude of branching, root hair formation and rooting depth. However, the underlying mechanisms and hormonal regulation of rice root elongation under low Pi conditions remain unclear.

Jasmonic acid (JA) is a phytohormone well known for its root growth inhibitory action (Noir et al. 2013). Besides regulating lateral root formation in *Arabidopsis* (Cai et al. 2014), JA is also implicated in regulating lateral root and root hair development in rice (Wang et al. 2002). Whilst the role of JA signaling in shaping root architecture is known, the link connecting JA and Pi signaling has remained elusive to date. As JAs are known to inhibit root growth, suppression of its levels and/or signaling under Pi limiting conditions may serve to enhance root growth and thus, Pi foraging capacity of plants.

JA signaling is classically described to be activated by external cues like wounding and various environmental challenges (Campos et al. 2014; Trang Nguyen et al. 2019; Ali and Baek 2020). JA signaling requires binding of JA-Ile (i.e., the bioactive form of JA) with the JA receptor (COI1). *COI1* (*CORONATINE INSENSITIVE 1*) encodes a F-box protein which is a part of SCF (SKP1/CUL1/F-box) E3 ubiquitin ligase complex (Devoto et al. 2002). Upon perception of JA-Ile, SCF^COI1^ targets JAZ repressors for ubiquitination and subsequent degradation via the 26S proteasome system (Chini et al. 2007; Thines et al. 2007). JAZ (JASMONATE ZIM-DOMAIN) proteins are transcriptional repressors of JA response pathways in plants. JAZ proteins directly bind to several transcription factors (TFs) and suppress their activity (Kazan and Manners 2012). These TFs include MYC2 which acts as a master regulator of JA-responsive genes. JAZ repressors along with co-repressors (NINJA and TOPLESS), repress MYC2 activity (Pauwels et al. 2010). Degradation of JAZ repressors relieve repression of MYC2 enabling it to activate expression of JA-responsive genes including *JAZ*s (Chini et al. 2007; Chung et al. 2009). Interestingly, several JAZ encoding genes have found to be differentially regulated by Pi deficiency in rice (Singh et al. 2015). From our previous microarray study of a low-Pi-tolerant and -sensitive rice genotype, we identified *OsJAZ11* as a low-Pi-responsive JAZ repressor that was significantly induced under Pi deficiency (Mehra et al. 2016).

In this study, we employed an *OsJAZ11* overexpression strategy to repress JA signaling in rice. We report that rice lines overexpressing *OsJAZ11* led to enhanced root growth under low Pi conditions in low-Pi-sensitive rice genotype. Our study also uncovered molecular mechanisms underlying the regulation of low Pi responses through *OsJAZ11*, providing new evidence connecting JA and phosphate signaling pathways.

## Materials and methods

### Plant material and treatments

Seeds of low-Pi-sensitive rice genotype, PB1 (*Oryza sativa* L.) were obtained from Indian Agricultural Research Institute (IARI), New Delhi, India. Seeds were surface sterilized and germinated as described previously (Pandey et al. 2017). Evenly germinated seeds were grown hydroponically in Yoshida growth medium containing 1 μM NaH_2_PO_4_ (−P) and 320 μM NaH_2_PO_4_ (+P) as described earlier (Pandey et al. 2017). Plants were harvested after 15 and 30 days of respective treatments for analysis. For +P to −P time-course experiments, rice seedlings were first raised under +P conditions for 7 days and subsequently transferred to −P conditions. Samples for quantitation of gene expression, Pi concentration, GUS expression and hormone concentration were collected at different time points. For MeJA sensitivity assays, 7-day-old seedlings were treated with 10 μM MeJA or DMSO (control) for 14 days. Plants were raised hydroponically under −P conditions throughout for 21 days. For MeJA treatment of *pOsJAZ11: GUS* transgenics, 4-day-old seedlings were treated with 1, 10, 50 and 100 μM MeJA for 1 h. Control seedlings were subjected to DMSO treatment.

For measuring JA response in *Arabidopsis* roots, Jas9-VENUS biosensor was used (Larrieu et al. 2015). Jas9-VENUS is used as a biosensor for perception of bioactive JA in plants (Larrieu et al. 2015). Jas9-VENUS signal undergoes rapid degradation with increase in bioactive JA. *Arabidopsis* seeds expressing Jas9-VENUS were surface sterilized and grown on LP (Low Phosphorus; 3 μM NaH_2_PO_4_) and HP (High Phosphorus; 312 μM NaH_2_PO_4_) for 10 days as described earlier (Bhosale et al. 2018). Confocal images were captured using Leica SP5 confocal microscope and raw integral density of fluorescence was quantified as described (Giri et al. 2018). For studying root phenotype of *Arabidopsis* (Col-0) under phosphate deficiency, seeds were surface sterilized and grown on LP and HP media for 10 days. Images were captured by Nikon DSLR 5200. Images were analyzed using ImageJ 1.46r as described earlier (Mehra et al. 2017).

### Plant phenotyping

Root lengths were measured manually with ruler. For dry biomass measurements, plant tissues were oven dried till constant weight. For quantitation of other root traits such as lateral numbers, lateral root length, seminal root numbers and seminal root length, images of 15-day-old roots were captured with Nikon DSLR 5200. Images were analyzed using ImageJ 1.46r as described earlier (Mehra et al. 2017). For average lateral root length, lateral root lengths of all laterals in a plant were measured and averaged. For lateral root length per cm of root, sum of lateral root lengths on each root was divided by length of root. For total lateral root number per plant, all laterals in each plant were counted. For lateral density, total number of laterals on each root was divided by the length of root. For seminal root length, lengths of all seminal roots in each plant were averaged. For total seminal root length, lengths of all seminal root in a plant were summed up. For seminal root number, total number of seminal roots per plant was counted. Means for all traits were obtained from data of four replicates. Statistical analysis was carried out using Student’s *t* test. Pairwise multiple comparisons were performed in Sigma Plot 12.0 using one-way ANOVA followed by Duncan’s multiple comparison test (*α* < 0.05).

### Expression analysis by quantitative real-time PCR

Total RNA was isolated using RNeasy Mini Kit (Qiagen) following manufacturer’s protocol. cDNA was synthesized using RevertAid First Strand cDNA Synthesis Kit (Thermo Fisher Scientific) as per manufacturer’s instructions. RT-qPCR was carried out on QuantStudio 3 Real-Time PCR system (Thermo Fisher Scientific) using PowerUp SYBR Green Master mix (Thermo Fisher Scientific). Gene-specific primers were designed using PRIMEREXPRESS version 2.0 (Applied Biosystems) as described previously (Singh et al. 2015). *Ubiquitin5* was used as endogenous control. Relative expression levels were computed using 2^−Δ(ΔCT)^ method. Primers used for RT-qPCR are listed in Table S1.

### Electrophoretic mobility shift assay (EMSA)

Protein coding region of OsPHR2 was cloned into NdeI and BamH1 restriction sites of pET28a expression vector using specific primers (Table S1). Recombinant OsPHR2-6XHIS protein was purified by Ni^2+^-affinity chromatography as described earlier (Mehra et al. 2017). For EMSA assay, 30 bp sense and antisense oligonucleotides (5′-GAGTCACATC**GGATATAC**ATACACATATTT-3′ and 5′-AAATATGTGTAT**GTATATCC**GATGTGACTC-3′) harboring the conserved *cis-*element P1BS (PHR1 Binding Sequence; bold) were designed from promoter region of *OsJAZ11*. For EMSA with mutated probes, complementary oligonucleotides with mutated P1BS *cis*-element (bold) were designed (5′-GAGTCACATC**TGCGCGAA**ATACACATATTT-3′) and 5′-AAATATGTGTAT**TTCGCGCA**GATGTGACTC-3′). Complementary oligonucleotides were annealed by heating at 95 °C for 10 min followed by subsequent cooling to room temperature. Double-stranded oligonucleotides were labeled with digoxigenin (DIG) at 3′ end using DIG Oligonucleotide 3′-End Labeling Kit as per manufacturer’s protocol (Roche Diagnostics). For assay, 50 ng of labeled promoter probe was incubated with different concentrations of recombinant OsPHR2 (100 ng, 200 ng, 300 ng and 400 ng) in 20 μl EMSA reaction buffer [(1 μg poly (dI-dC), 30 mM KCl, 15 mM HEPES (pH 8.0), 0.02 mM DTT, 1 mM MgCl_2_, 0.2 mM EDTA and 0.6% glycerol)]. For, competition EMSA, 200-fold excess unlabeled double-stranded oligonucleotide was added to the reaction. All reactions were incubated at 20 °C for 30 min followed by electrophoresis on 10% non-denaturing polyacrylamide gel in 0.5X TBE running buffer at 4 °C. Samples were transferred to positively charged nylon membrane (Amersham Biosciences) and detected using DIG Nucleic Acid Detection Kit according to manufacturer’s instructions (Roche Diagnostics).

### Pi content and JA measurements

Total and soluble Pi measurements were performed as described earlier (Mehra et al. 2019). Jasmonic acid levels were measured in root tissues as described previously (Lin et al. 2019). Briefly, 100 mg of roots was weighed and frozen immediately in liquid nitrogen. Tissues were homogenized in 1 ml of 1X PBS buffer pH 7.4. Homogenates were centrifuged and supernatant fraction was collected. Jasmonic acid levels were subsequently measured using quantitative sandwich enzyme-linked immunosorbent assay (ELISA) kit (MyBioSource, San Diego, CA, USA) according to the manufacturer's protocol. All measurements were made in six independent biological replicates.

### Vector construction and raising of transgenics

For generating *OsJAZ11* overexpression construct, full-length cDNA of *OsJAZ11* (LOC_Os03g08320) was cloned under maize ubiquitin promoter (*pZmUbi1*) in Gateway-compatible binary vector pANIC6B (Mann et al. 2012). For silencing of *OsJAZ11*, 350 bp region of *OsJAZ11* was amplified using gene-specific primers and cloned in RNAi vector, pANIC8B (Table S1). For generating *OsJAZ11-GUS* and *OsJAZ11ΔC-GUS* transgenics, *OsJAZ11* CDS and *OsJAZ11ΔC* (OsJAZ11 CDS with 57 a.a. deleted at C-terminal region) were cloned under *CaMV35S* promoter in pCAMBIA1301 vector. For generating *pOsJAZ11: GUS* transcriptional reporters, 1.5 kb promoter region of *OsJAZ11* upstream of start codon was amplified. Promoter was cloned upstream of *GUS* (*β-glucuronidase*) reporter sequence into destination vector, pMDC163 vector. All constructs were transformed in to low-Pi-sensitive rice genotype PB1 by *Agrobacterium*-mediated transformation (Mehra et al. 2017). Positive transformants were selected on hygromycin (50 μg/ml) and screened by RT-qPCR. All experiments were conducted in T3 homozygous lines. All primers are listed in Table S1.

### In vivo repressor assay and analysis of GUS activity

In vivo repressor assay of OsJAZ11 was performed as described previously (Singh et al. 2020). Briefly, roots of 15-day-old *OsJAZ11-GUS* and *OsJAZ11ΔC-GUS* transgenics were treated with 100 μM MeJA with or without the proteasome inhibitor 100 μM MG132 for 1 h. For control, seedlings were treated with DMSO alone. Histochemical GUS staining was performed as described earlier (Mehra et al. 2019). Images were captured with a Zeiss stereo zoom microscope. Quantitation of GUS signals were performed fluorometrically as described earlier (Mehra et al. 2019).

### Yeast two-hybrid assays

Yeast two-hybrid assays were carried out using Matchmaker Gold Yeast two-hybrid system (Clontech, Mountain View, CA, USA) according to manufacturer’s protocol. Protein coding region of target genes was cloned into pENTR vector and then mobilized into appropriate destination vectors; bait vector (BD, pGBKT7) or prey vector (AD, pGADT7) by GATEWAY technology (Invitrogen). AD and BD clones were co-transformed in yeast strain, Y2H Gold (Clontech) and selected on Double drop-out (DDO) medium, i.e., SD/-Leu/-Trp (-LT). For interactions assays, co-transformed colonies were suspended in minimal medium (-LT) and 5 μl of suspended cells was spotted on agar plates containing triple drop-out (TDO) medium SD/-Leu/-Trp/-His (-HLT), quadruple drop-out (QDO) medium SD/-Leu/-Trp/-His/-Ade (-AHLT) and quadruple drop-out medium supplemented with 40 μg/ml X-α-gal and 200 ng/ml Aureobasidin A (SD/-Leu/-Trp/-His/-Ade/X-Gal/Aureobasidin A). Plates were incubated at 30 °C for 3–4 days. Images were captured with Nikon DSLR 5200. All gene-specific primers used for cloning are listed in Table S1.

### In vitro pull-down assay

Coding regions of *OsJAZ11* and *OsSPX1* were cloned into appropriate restriction sites in expression vectors pGEX4T1 and pET28a, respectively to overexpress recombinant protein OsJAZ11-GST and OsSPX1-6XHIS. Empty vector and prepared vector constructs were independently transformed and induced in *E. coli* strain BL21(DE3)pLysS as described earlier (Pandey et al. 2017). Bacterial proteins were isolated at 4 °C by suspending cells in lysis buffer (1X PBS pH 7.4 containing 0.5 mM DTT, 1 mM phenylmethylsulfonyl fluoride, 1X bacterial Protease Inhibitor Cocktail, and 1 mg/ml lysozyme). Suspended cells were incubated with lysis buffer for 1 h followed by brief sonication. Cell suspension was subjected to centrifugation to separate supernatant fraction containing recombinant proteins. OsSPX1-6XHIS protein was subsequently purified from supernatant fraction by Ni^2+^-affinity chromatography (Immobilized metal ion chromatography, IMAC) as described earlier (Mehra and Giri 2016).

While, OsJAZ11-GST and GST proteins were immobilized on glutathione-agarose beads (Sigma-Aldrich) according to the manufacturer’s protocol. Recombinant purified OsSPX1-6XHIS protein was incubated with immobilized OsJAZ11-GST and GST proteins for 4 h at 4 °C with gentle shaking. After 4 h, agarose beads were washed twice with 1X PBS. Bound proteins were eluted with 30 mM reduced glutathione (pH 8.0). Eluted proteins were separated on 12% SDS−PAGE and subjected to immunoblotting as described earlier (Mehra et al. 2017). GST and OsJA11-GST were detected using rabbit anti-GST primary antibody (Sigma-Aldrich) and horseradish peroxidase-labeled goat anti-rabbit IgG as secondary antibody (Sigma-Aldrich). While, OsSPX1-6XHIS proteins were detected with mouse anti-HIS primary antibody and horseradish peroxidase-labeled goat anti-mouse IgG as secondary antibody (Sigma-Aldrich). Primary and secondary antibodies were used in 1:1000 and 1:10,000 dilution, respectively. 6XHIS and GST signals were detected using ECL Prime western blotting detection kit (GE Healthcare Biosciences) as per manufacturer’s instructions.

### Identification of OsJAZ11 interacting proteins from plant

OsJAZ11-GST protein was extracted and immobilized on glutathione-agarose beads as described above. Total protein from 30-day-old *OsJAZ11* overexpression lines was isolated using extraction buffer (1X PBS pH 7.4 containing 0.5 mM DTT, 1 mM phenylmethylsulfonyl fluoride, 1X plant Protease Inhibitor Cocktail). Extracted protein was incubated with recombinant OsJAZ11-GST protein bound to glutathione-agarose beads at 4 °C overnight. Beads were subsequently washed thrice with 1X PBS and OsJAZ11-bound protein complexes were eluted with 30 mM reduced glutathione (pH 8.0). Eluted proteins were identified by LC–MS using Exactive™ Plus Orbitrap Mass Spectrometer (Thermo Fisher Scientific).

### OsPHR2 activity inhibition assay

Thirty bp sense and antisense oligonucleotides (Table S1) harboring the P1BS element were designed from promoter of *OsSPX2*. Complementary oligonucleotides were annealed and DIG-labeled as summarized above. 50 ng of the labeled probe was used for EMSA assay. For inhibition assay, 0.7 μg OsPHR2-6XHIS protein was incubated with 7.5 μg OsSPX1 protein in EMSA reaction buffer in the presence of 15 mM NaH_2_PO_4_. To this reaction, 7.5 μg of OsJAZ11-GST or GST (negative control) was added for analyzing the effect of OsJAZ11 on OsSPX1-mediated inhibition of OsPHR2. All reactions were incubated at 20 °C for 1 h and electrophoresed on 8% non-denaturing polyacrylamide gel in 0.5X TBE running buffer at 4 °C. Blotting and probe detection were carried out as described above in EMSA method section.

## Results

### *OsJAZ11* is a JA-responsive gene

In rice, 15 *JAZ* genes have been identified so far (Singh et al. 2015). *OsJAZ11* (LOC_Os03g08320) locus transcribes a single transcript that encodes for a 209 a.a. protein containing conserved TIFY and Jas motif (Supplementary Fig. S1a). As described in previous phylogenetic analysis of identified JAZ proteins, OsJAZ11 shares close homology with few other rice JAZ proteins (OsJAZ9, 10, 12, 13, 14, and 15) and groups together with them in Clade IIc (Singh et al. 2015). JA treatment assays revealed that *OsJAZ11* is upregulated within 0.25 h of JA treatment (Supplementary Fig. S1b). An *OsJAZ11* promoter driven GUS transcriptional reporter also exhibited upregulation when exposed to MeJA (Supplementary Fig. S1c). These results confirm that *OsJAZ11* is highly JA responsive.

### *OsJAZ11* is induced by low Pi

**Fig. 1:**
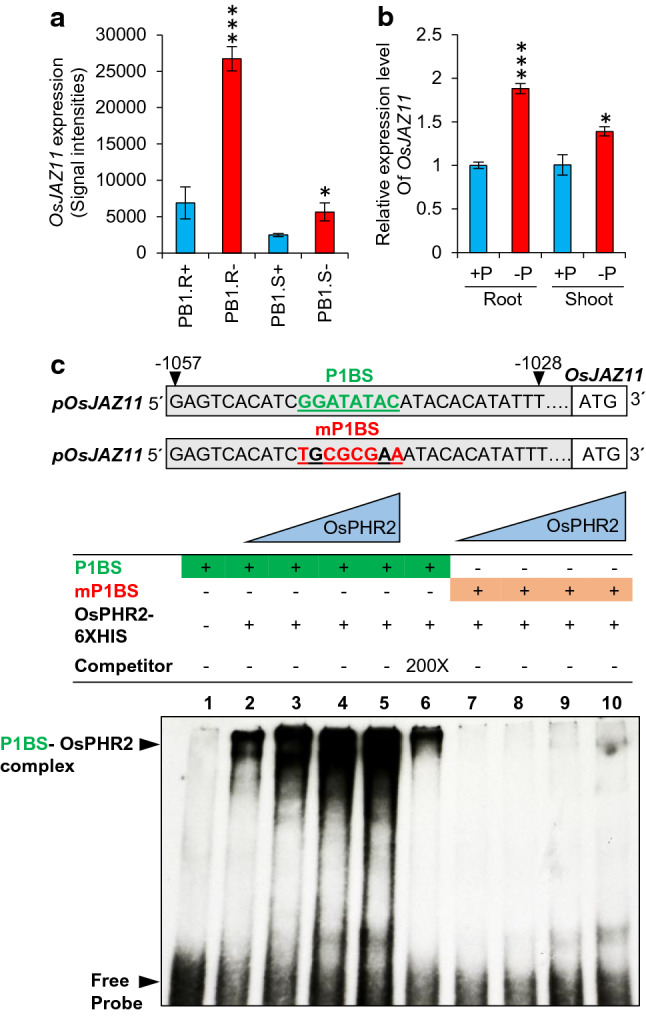
*OsJAZ11* is induced by Pi deficiency. **a** Expression pattern of *OsJAZ11* in shoot (PB1.S) and root (PB1.R) of low-Pi-sensitive rice genotype PB1 under +P and −P conditions. Signal intensities were retrieved from previous microarray dataset (Mehra et al. 2016) submitted in Gene Expression Omnibus (GEO) database (accession no. GSE74795). **b** RT-qPCR of *OsJAZ11* in roots and shoots of 30-day-old PB1 plants under +P (320 μM NaH_2_PO_4_) and −P (1 μM NaH_2_PO_4_) conditions. Each bar is the mean of three independent replicates with standard error. Significant differences between +P vs −P treatments were evaluated by Student’s *t* test. Asterisks * and *** indicate *P* values, ≤ 0.05 and 0.001, respectively (*n* = 3). **c** EMSA assays showing physical interaction of *OsJAZ11* promoter with OsPHR2-6XHIS at P1BS *cis*-element. Interactions were performed using 50 ng of DIG-labeled *OsJAZ11* promoter probe (− 1057 to − 1028 bp) flanking P1BS (lanes 1–6). Interactions were carried out with different concentrations of OsPHR2-6XHIS (100 ng, 200 ng, 300 ng and 400 ng in lane 2, 3, 4 and 5, respectively). In lane 6, for competition assays, 200 ng of OsPHR2-6XHIS and 200-fold excess of unlabeled *OsJAZ11* promoter was used. In lanes 7–10, 50 ng of *OsJAZ11* promoter probe was used with mutations in P1BS element (mP1BS). 100 ng, 200 ng, 300 ng and 400 ng of OsPHR2-6XHIS was used in lanes 7, 8, 9 and 10, respectively

Previously, we performed microarray-based transcriptional profiling of rice genotypes under +P and −P conditions (Mehra et al. 2016). We reanalyzed this dataset to identify differentially expressed rice *JAZ* genes under low Pi. Our analysis revealed that *OsJAZ11* gene undergoes significant upregulation in rice roots under −P (1 μM NaH_2_PO_4_) compared to +P (320 μM NaH_2_PO_4_) conditions (Fig. 1a). We validated this microarray-based result using RT-qPCR and found significant upregulation of *OsJAZ11* in roots exposed to 30 days of low Pi treatment (Fig. 1b). To better understand the effect of low Pi on its expression, we performed a detailed temporal expression profiling of *OsJAZ11* in rice roots after transferring rice plants from +P to −P conditions. After 30 min of −P treatment, *OsJAZ11* exhibits a 4.9-fold increase in expression, which declines to 0.5-fold at 1 h. Notably, there is no change in systemic Pi concentrations at these time points (Supplementary Fig. S2). This implies that *OsJAZ11* is responsive to early events of low Pi sensing and signaling due to local alterations in exogenous Pi concentrations surrounding the root tip. After prolonged Pi deficiency (14–21 days of −P treatment), when the Pi pool in plant tissues depletes significantly, expression of *OsJAZ11* steeply increases by up to 7.4-fold. Our temporal expression analysis reveals potential roles of *OsJAZ11* during both local as well as systemic Pi signaling.

Previous transcriptomic studies have reported that many PSI (Phosphate Starvation-Inducible) genes are transcriptionally activated by MYB transcription factor, PHOSPHATE STARVATION RESPONSE REGULATOR 1 (PHR1). Both *Arabidopsis* AtPHR1 and its rice homologue OsPHR2 bind to the conserved *cis*-element P1BS (PHR1 Binding Sequence; GNATATNC) in promoters of low-Pi-responsive genes (Zhou et al. 2008). To test whether OsPHR2 also regulates the expression of *OsJAZ11* under −P conditions, we surveyed a 1.5 kb promoter and 5′ untranslated region. Our search identified one P1BS element located 1047 bp upstream of the *OsJAZ11* ATG. To confirm physical binding of OsPHR2 to the identified P1BS element, EMSA assay was performed using a 30 bp DIG-labeled *OsJAZ11* promoter probe harboring the P1BS element (Fig. 1c). Our study established the physical binding of OsPHR2 with *OsJAZ11* promoter. The strength of binding also increased with increasing concentrations of recombinant 6XHIS-OsPHR2. Performing EMSA with 200X unlabeled probe (competitor) and *OsJAZ11* labeled probe with mutated P1BS (mP1BS) exhibited reduced and no binding with recombinant OsPHR2 protein, respectively (Fig. 1c). This confirmed specific binding of OsPHR2 to the P1BS *cis*-element in the OsJAZ*11* promoter. These results indicate that an OsPHR2-dependent low Pi response pathway induces expression of *OsJAZ11*.

### *OsJAZ11* overexpression increased total phosphorus (P) uptake

**Fig. 2:**
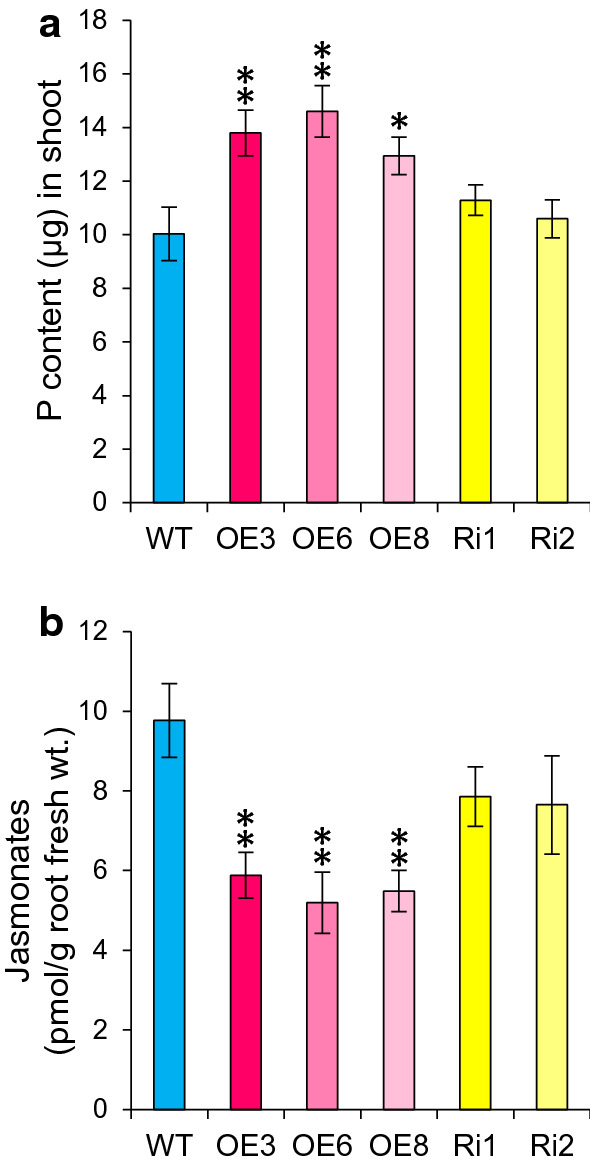
Total phosphorus and JA content of WT and *OsJAZ11* transgenics. **a** Total phosphorus content in shoots of WT and *OsJAZ11* transgenics. Each bar represents mean of ten independent replicates with standard error (*n* = 10). **b** JA content/g root fresh weight of WT and *OsJAZ11* transgenics. Plants were grown under –P conditions for 30 days. Each bar represents mean of six independent replicates with standard error (*n* = 6). Significant differences between WT and transgenics were evaluated by Student’s *t* test. Asterisks; * and ** indicate *P* values, ≤ 0.05 and 0.01, respectively

To explore the biological roles of *OsJAZ11*, overexpression (OE) and RNAi (Ri) lines of *OsJAZ11* were generated in rice using the *ZmUbi1* promoter (Supplementary Fig. S3). All OE lines exhibited 400- to 800-fold overexpression of *OsJAZ11* compared to wild type (WT), while *OsJAZ11* expression was reduced 12–23% in the RNAi lines (Supplementary Fig. S3). To assess the impact of *OsJAZ11* on P uptake, total P content was quantitated in WT and transgenics after 30 days of +P and −P treatments. Notably, shoots and roots of OE lines showed increased P content (30–45% and 43–68%, respectively) as compared to WT under −P conditions (Fig. 2a, Supplementary Fig. S4b). Similarly, under +P conditions, significant increase in total P content was observed in OE lines as compared to WT (Supplementary Fig. S4a and c). However, no differences in P content were observed between WT and RNAi lines (Fig. 2a, Supplementary Fig. S4). These results reveal a positive role for *OsJAZ11* in imparting low P tolerance to rice through enhanced P uptake.

We next investigated the expression of low-Pi-responsive marker genes in WT and *OsJAZ11* transgenics. Under −P conditions, OE lines showed significantly reduced expression of Pi transporters (*OsPT8/9*) and other PSR (Phosphate Starvation Response) genes such as *OsIPS1*, *OsGDPD5*, *OsMGD3*, *OsPAP3b* and *OsPAP10a* (Supplementary Fig. S5). In contrast, RNAi lines showed enhanced expression of most PSR genes. These results reveal suppressed PSR in OE lines compared to WT under Pi deficiency.

### *OsJAZ11* overexpression suppressed JA biosynthesis and signaling in rice

OsJAZ11 is an important component of the rice JA response pathway; hence, alterations in its levels may in/directly influence JA biosynthesis and/or signaling in rice. To explore such a possibility, JA levels were measured in roots of OE and RNAi lines in 30-day-old rice seedlings grown under −P conditions. Interestingly, all OE lines exhibited a significant drop (40–47% decrease) in endogenous JA levels compared to WT in rice root under −P (Fig. 2b). On the contrary, no significant changes in JA levels were observed in RNAi lines compared to WT. In agreement with these findings, genes encoding JA biosynthesis enzyme allene oxide synthase (AOS); *OsAOS1* and *OsAOS2* were significantly downregulated in roots of OE lines compared to WT (Supplementary Fig. S6). No change in expression of *OsAOS1/2* was observed in RNAi lines. We also measured expression of several JA signaling genes, *MYC2* and other *JAZ* repressors in transgenic lines. Interestingly, *OsMYC2*, *OsJAZ8* and *OsJAZ9* were significantly downregulated in OE lines compared to WT. On the other hand, *OsJAZ8* and *OsJAZ9* were significantly upregulated in RNAi lines (Supplementary Fig. S6). Collectively, our results indicate a suppression of JA biosynthesis and signaling in OE lines compared to WT. Hence, overexpression of JAZ repressor *OsJAZ11* appears to significantly perturb JA signaling in rice.

### *OsJAZ11* overexpression enhances rice root growth

**Fig. 3:**
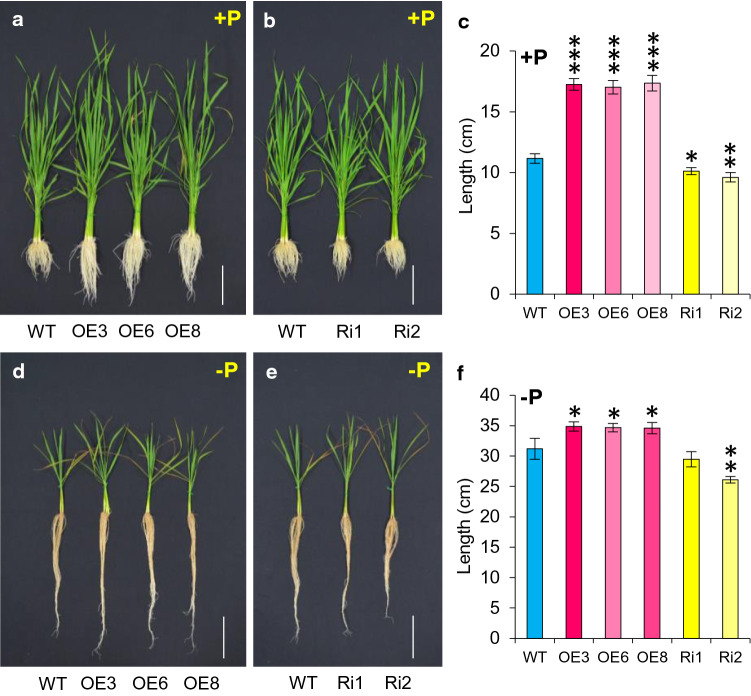
Overexpression transgenics of *OsJAZ11* developed longer roots than WT. Root phenotype of 30-day-old *OsJAZ11* overexpression (OE) lines (**a**, **d**) and silencing RNAi (Ri) lines (**b**, **e**) compared to WT under +P (**a**, **b**) and −P (**d**, **e**) conditions. For imaging, three representative plants of each line were stacked together. Scale bar = 10 cm. Quantitation of root lengths of WT, OE and Ri lines under +P conditions (**c**) and −P (**f**) conditions. Each bar represents mean of ten biological replicates with standard error. Significant differences between WT and transgenics were evaluated by Student’s *t* test. Asterisks; *, ** and *** indicate *P* values, ≤ 0.05, 0.01 and 0.001, respectively (*n* = 10)

We next investigated whether suppressed JA biosynthesis and signaling in OE lines is sufficient to alleviate the root inhibitory effect of JA. Thus, we investigated the effect of *OsJAZ11* on root growth under Pi deficiency. OE and RNAi lines were grown hydroponically under +P and −P conditions for 30 days. Root length was measured after 15 and 30 days of treatments. Notably, OE lines showed 11–14% increase in root length as compared to WT under −P (Fig. 3d and f; Supplementary Fig. S7). Under +P conditions, root length was not affected significantly after 15 days of treatment (Supplementary Fig. S7). However, after 30 days, a significant increase in root length was observed in OE lines compared to WT even under +P conditions (Fig. 3a and c). We also quantified the effect of increased root length on total root biomass of OE lines after 30 days of treatment. Expectedly, OE lines exhibited 31–47% increase in root dry weight compared to WT under −P conditions (Supplementary Fig. S8b). Under +P, 1.5 to twofold increases in root dry weight were observed in OE lines (Supplementary Fig. S8a). Higher root biomass also promoted an increased total shoot dry weight in OE lines as compared to WT especially under low Pi stress conditions (Supplementary Fig. S8b). Contrary to this, RNAi lines displayed a significant reduction in root length both under +P and −P conditions (Fig. 3b, c and e, f; Supplementary Fig. S7). However, decreased root length did not induce a significant change in root or shoot biomass in RNAi lines under +P or −P conditions (Supplementary Fig. S8).

**Fig. 4:**
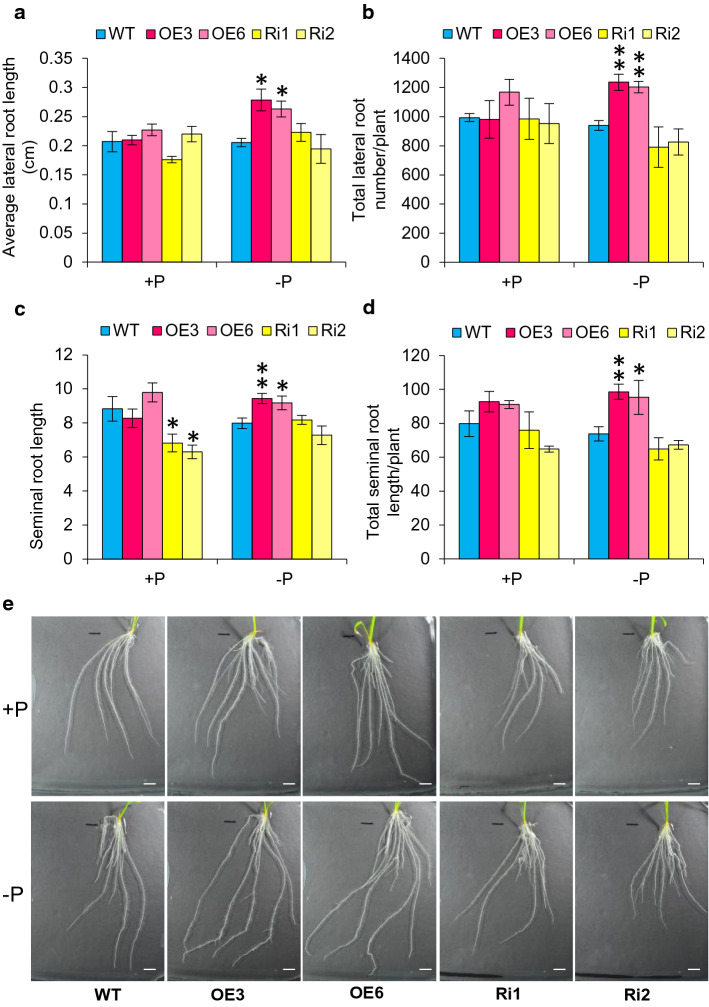
Effect of *OsJAZ11* on root traits. **a–d** Average lateral root length, total number of lateral roots per plant, seminal root length and total seminal root length per plant in 15-day-old WT and *OsJAZ11* transgenics under +P and −P conditions. **e** Root phenotypes of 15-day-old WT and *OsJAZ11* transgenics under +P and −P conditions. White line at bottom of each image represents scale of 1 cm. Each bar represents mean from four replicates with standard error. Significant differences between WT and transgenics were evaluated by Student’s *t* test. Asterisks; * and ** indicate *P* values, ≤ 0.05 and 0.01, respectively (*n* = 4)

Higher concentrations of JA are reported to inhibit seminal root elongation and lateral root development in rice (Wang et al. 2002; Singh et al. 2020). Therefore, we also investigated the impact of *OsJAZ11* on seminal and lateral root growth. Our analysis revealed significant increase in seminal root length in OE lines under −P (Fig. 4c and e). Total seminal root growth per plant (measured as sum total of seminal root length/plant) in OE lines was 1.3-fold higher than in WT under Pi deficiency (Fig. 4d). In RNAi lines, no significant reduction was observed in average and total seminal root length under low Pi stress (Fig. 4c and d). No significant differences in seminal root length were observed between WT and OE lines under +P conditions (Fig. 4c and d). However, RNAi lines showed reduction in seminal root length under +P conditions (Fig. 4c). Overexpression of *OsJAZ11* enhanced average lateral root length in OE lines under −P (Fig. 4a). Whilst there was no change in lateral density (number of laterals/cm of main root) (Supplementary Fig. S9), but due to increased seminal root length in OE lines, total number of laterals per plant was significantly higher in OE lines compared to WT under −P (Fig. 4b). In all transgenics, other RSA (Root System Architecture) traits such as lateral length per cm of primary root and seminal root number were similar to WT under both +P and −P conditions (Supplementary Fig. S9). Taken together, our findings suggest that overexpression of *OsJAZ11* suppressed JA signaling in rice roots, leading to enhanced root proliferation in OE lines compared to WT, especially under −P conditions. These results also reveal that enhanced root growth in OE lines led to higher Pi acquisition which ultimately suppressed the induction of PSR genes in OE lines under Pi deficiency.

### The OsJAZ11 C-terminal region is required to regulate root growth in OE lines

JAZ repressors contain a conserved Jas motif at their C-terminal which is necessary for 26S proteasome-mediated degradation via the SCF^COI1^ complex (Chini et al. 2007; Melotto et al. 2008). Additionally, the Jas motif is required for JA-dependent JAZ–MYC2 interactions. MYC2 is a basic helix–loop–helix (bHLH) master transcription factor that regulates diverse JA responses. To determine the functional significance of the C-terminal region of OsJAZ11 for regulating root elongation, we overexpressed OsJAZ11-GUS and OsJAZ11ΔC-GUS (with deleted C-terminal) fusion proteins under *CaMV35S* promoter in rice (Supplementary Fig. S10a and b). All translational reporter lines exhibited significant overexpression of *OsJAZ11* at both transcript and protein levels (Supplementary Fig. S10b). Treatment of *OsJAZ11-GUS* transgenics with 100 μM MeJA greatly reduced the GUS reporter signal. In contrast, application of 100 μM MG132, a potent 26S proteasome inhibitor, inhibited MeJA-dependent degradation of OsJAZ11-GUS (Supplementary Fig. S10c and e). However, stable GUS reporter signals were observed in *OsJAZ11ΔC-GUS* transgenic roots even after MeJA treatment (Supplementary Fig. S10d and f). This reveals that the OsJAZ11 protein undergoes 26S proteasome-mediated degradation through its C-terminal region and therefore, *OsJAZ11ΔC-GUS* transgenics behave as constitutive repression lines. These results also establish the *in vivo* repression activity of OsJAZ11 in response to MeJA.

**Fig. 5:**
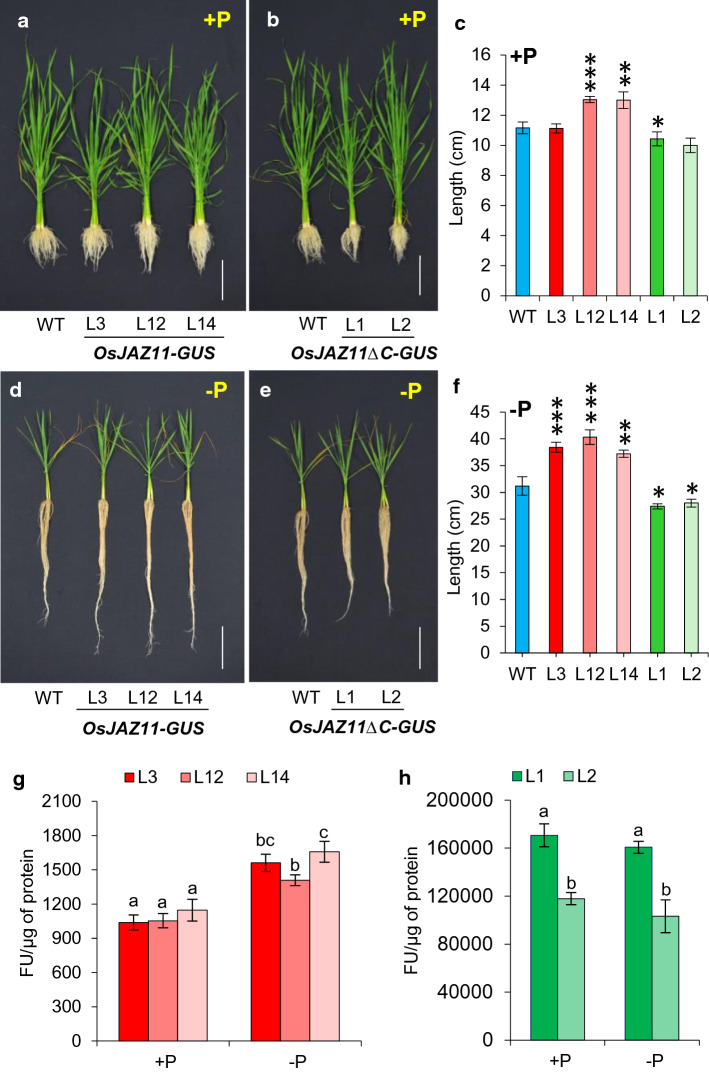
C-terminal domain of OsJAZ11 regulates root growth. Plant phenotype of 30-day-old *OsJAZ11-GUS* (**a**, **d**), *OsJAZ11ΔC-GUS* (**b**, **e**) translational reporter lines and WT under +P (**a**, **b**) and −P (**d**, **e**) conditions. For imaging three representative plants of each line were stacked together. Scale bar = 10 cm. **c** Quantitation of root lengths of WT, *OsJAZ11-GUS* and *OsJAZ11ΔC-GUS* lines under +P conditions. **f** Quantitation of root lengths of WT, *OsJAZ11-GUS* and *OsJAZ11ΔC-GUS* lines under −P conditions. Each bar represents mean of ten biological replicates with standard error. Significant differences between WT and transgenics were evaluated by Student’s *t* test. Asterisks; *, ** and *** indicate *P* values, ≤ 0.05, 0.01 and 0.001, respectively (*n* = 10). **g-h** Fluorometric quantitation of GUS activity in 30-day-old *OsJAZ11-GUS* (L3, L12, L14) and *OsJAZ11ΔC-GUS* (L1, L2) transgenics under +P and –P conditions. Each bar represents mean of six biological replicates with standard error. Different letters on top of each bar denotes significant differences determined by one-way ANOVA followed by Duncan’s multiple comparison test (*α* < 0.05) (*n* = 6)

Next, we analyzed the root phenotypes of *OsJAZ11-GUS* and *OsJAZ11ΔC-GUS* transgenics under +P and −P conditions. Similar to overexpression transgenics (with *ZmUbi1* promoter), *OsJAZ11-GUS* transgenics showed a significant increase in root length after 15 and 30 days of −P treatment as compared to WT (Fig. 5d and f; Supplementary Fig. S11d and f). Under +P conditions also, a significant increase in root length was observed in *OsJAZ11-GUS* lines after 30 days of treatment (Fig. 5a and c). These results provide additional evidence to further confirm the important role of *OsJAZ11* in regulating rice root length. Interestingly, constitutive repression transgenics (*OsJAZ11ΔC-GUS*) exhibited a 17- 20% and 10–12% reduction in root length compared to WT after 15 and 30 days of −P treatments, respectively (Fig. 5e and f; Supplementary Fig. S11e and f). Significant reduction in root lengths was also observed in *OsJAZ11ΔC-GUS* lines under +P conditions (Fig. 5b and c; Supplementary Fig. S11b and c). Moreover, *OsJAZ11-GUS* transgenics exhibited longer seminal roots as compared to WT under −P conditions (Supplementary Fig. S12). Due to increased root length, *OsJAZ11-GUS* lines also accumulated significantly higher root and shoot biomass as compared to WT under −P conditions (Supplementary Fig. S13). On the other hand, *OsJAZ11ΔC-GUS* lines showed significant decrease in seminal root length under both +P and −P conditions (Supplementary Fig. S12). Collectively, our results suggest that C-terminal region of OsJAZ11 containing the Jas motif is important for regulating root elongation in overexpression lines.

### Deletion of C-terminal region in *OsJAZ11ΔC-GUS* transgenics reduced Pi uptake

We next investigated whether there was any impact of reduced root length on Pi uptake in *OsJAZ11ΔC-GUS* transgenics. For this, seven-day-old +P grown *OsJAZ11-GUS* and *OsJAZ11ΔC-GUS* transgenics were transferred to −P liquid medium and Pi content was subsequently determined at different time points up to 14 days of −P treatment (Supplementary Fig. S14). Notably, *OsJAZ11ΔC-GUS* lines showed reduced Pi content per unit of root biomass as compared to *OsJAZ11-GUS* transgenics nearly at all time-points. This comparative analysis reveals compromised Pi uptake efficiency in constitutive repression (*OsJAZ11ΔC-GUS*) lines as compared to full-length *OsJAZ11* overexpression transgenics owing to reduced root growth in *OsJAZ11ΔC-GUS* transgenics. Further, Pi content estimation in 30-day-old translational reporters also confirmed higher Pi accumulation in OsJAZ*11-GUS* lines as compared to *OsJAZ11ΔC-GUS* lines (Supplementary Fig. S15).

### Pi deficiency leads to an accumulation of OsJAZ11 in rice

JA signaling results in proteasome-mediated degradation of JAZ proteins. Therefore, we determined the effect of Pi deficiency on OsJAZ11 protein levels using the translational reporter lines. The GUS reporter signals were quantitated in 30-day-old +P and −P grown *OsJAZ11-GUS* and *OsJAZ11ΔC-GUS* transgenics. Similar to mRNA transcripts, significant accumulation of OsJAZ11 protein was observed in *OsJAZ11-GUS* reporter lines under −P as compared to +P conditions (Fig. 5g). In contrast, OsJAZ11ΔC-GUS protein levels were stable in *OsJAZ11ΔC-GUS* transgenics under +P and −P conditions (Fig. 5h). These results suggest that Pi deficiency leads to OsJAZ11 protein accumulation by suppressing 26S proteasome-mediated OsJAZ11 degradation.

### *OsJAZ11-GUS* transgenics exhibit altered JA sensitivity

As MeJA treatment induces OsJAZ11 protein turnover, overexpression of *OsJAZ11* may alter JA sensitivity in transgenic plants. Therefore, we investigated the JA sensitivity of *OsJAZ11* transgenics by monitoring their root length phenotypes after 14 days of MeJA treatment (Supplementary Fig. S16). As expected, overexpression lines exhibited reduced sensitivity to MeJA treatments compared to WT, whereas RNAi lines and *OsJAZ11ΔC-GUS* transgenics featured increased sensitivity to MeJA (Supplementary Fig. S16). Our results suggest that overexpression of *OsJAZ11* leads to suppression of JA signaling, whereas deletion of the C-terminal renders JA hypersensitivity to *OsJAZ11ΔC-GUS* transgenics.

### OsJAZ11 interacts with components of JA signaling

OsMYC2 is an important regulator of JA signaling in rice. Yeast two-hybrid assays confirmed the interaction of OsJAZ11 with OsMYC2, revealing a potential role for OsMYC2 regulating OsJAZ11-mediated JA signaling (Supplementary Fig. S17). Y2H studies also revealed an interaction between OsJAZ11 and transcriptional co-repressor OsNINJA1 (Novel INteractor of JAZ) (Supplementary Fig. S17). NINJA acts as an adaptor protein that recruits the TOPLESS (TPL) transcriptional co-repressor through its TPL-binding EAR repression motif (Pauwels et al. 2010). To identify other important OsJAZ11 interactors, we also performed in vitro pull-down assays from OsJAZ11 OE seedlings using recombinant OsJAZ11-GST (Supplementary Fig. S18). LC–MS analysis of eluted OsJAZ11 protein complexes led to the identification of several TPLs (TPL1/2/3) (Table S2). Our results suggest that OsJAZ11-NINJA-TPL acts as a functional JA signaling repressor complex in rice that suppresses the function of bHLH transcription factor, OsMYC2.

### OsJAZ11 interacts with OsSPX1 to regulate Pi homeostasis

**Fig. 6:**
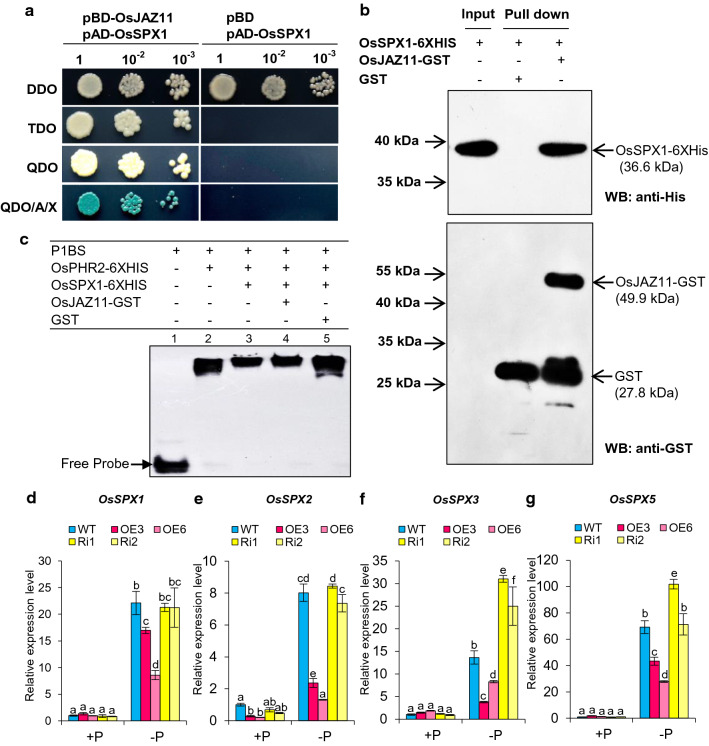
OsJAZ11 interacts with OsSPX1 to regulate Pi homeostasis. **a** Yeast two-hybrid assay showing interaction of OsJAZ11 (pBD-OsJAZ11) with OsSPX1 (pAD-OsSPX1). Serial dilutions of Y2H Gold strain co-transformed with AD (prey) and BD (bait) clones were spotted on DDO (SD-Leu/-Trp), TDO (SD- Leu/-Trp/-His), QDO (SD- Leu/-Trp/-His/-Ade) and QDO/A/X (QDO + Aureobasidin A + X-α-Gal). **b** GST pull-down assay showing interaction of OsJAZ11-GST and OsSPX1-6XHis. GST (negative control) and OsJAZ11-GST were immobilized on glutathione-agarose beads and incubated with OsSPX1-6XHIS (input protein). Pulled-down complexes were detected by immunoblotting using anti-GST and anti-HIS antibodies. **c** EMSA assay showing no effect of OsJAZ11 on OsSPX1-mediated inhibition of OsPHR2 binding to P1BS. OsPHR2-6XHIS (0.7 μg) and 50 ng DIG-labeled P1BS probe was incubated with either 7.5 μg SPX-6HIS (lane 3), SPX-6XHIS + 7.5 μg OsJAZ11-GST (lane 4) or SPX-6XHIS + 7.5 μg GST (lane 5). **d–g** Relative expression levels of *OsSPX1*, *OsSPX2*, *OsSPX3* and *OsSPX5* in roots of 30-day-old WT and *OsJAZ11* transgenics. Each bar is the mean of three independent replicates with standard error. Different letters on top of each bar denote significant differences determined by one-way ANOVA followed by Duncan’s multiple comparison test (*α* < 0.05) (*n* = 3)

To identify important components of Pi signaling pathways regulated by OsJAZ11, we analyzed the interaction of OsJAZ11 with key regulators of Pi homeostasis and signaling in rice. We examined the interaction of OsJAZ11 with OsPHO1.2, OsPHO2, OsSPX1, OsSPX3 using yeast two-hybrid assays (Fig. 6a; Supplementary Fig. S17). Of these, we observed strong interaction of OsJAZ11 with OsSPX1 in yeast cells (Fig. 6a). To support further that OsSPX1 is a direct target of OsJAZ11, we performed pull-down experiments using purified recombinant OsJAZ11-GST and protein extracts of bacteria overexpressing recombinant OsSPX1-6XHis protein. As shown in Fig. 6b, the results from pull-down experiments were consistent to those in yeast.

SPX proteins are named after SYG1 (suppressor of yeast gpa1), Pho81 (CDK inhibitor in yeast PHO pathway), and XPR1 (xenotropic and polytropic retrovirus receptor). OsSPX1 is involved in Pi sensing and acts as a negative regulator of Pi deficiency responses in rice. Under sufficient Pi conditions, OsSPX1 binds to OsPHR2 through its SPX domain and inhibits binding of OsPHR2 to P1BS elements (Wang et al. 2014). Therefore, we determined the impact of the OsJAZ11–OsSPX1 interaction on OsPHR2’s ability to bind to P1BS elements. To assess this, we performed EMSA assays using a DIG-labeled *OsSPX2* promoter containing a P1BS motif. OsPHR2 exhibited physical binding to the P1BS motif in EMSA assay (Fig. 6c). In agreement with previous reports, the amplitude of OsPHR2 binding reduced in the presence of OsSPX1-6XHIS, consistent with its reported inhibitory regulatory effect. However, the addition of purified recombinant OsJAZ11-GST did not appear to impact OsSPX1-mediated inhibition of OsPHR2 activity. Our results suggest that the OsJAZ11–OsSPX1 interaction may regulate Pi homeostasis in a PHR2-independent manner in rice. The expression of *SPX* family members (except *OsSPX4* which is not Pi inducible) was significantly reduced in *OsJAZ11* overexpression lines compared to WT under −P conditions (Fig. 6d–g; Supplementary Fig. S19). Conversely, in RNAi lines, *SPX* expression was either unchanged or higher compared to WT (Fig. 6). Reduced expression of *SPX* family members by OsJAZ11 may function to enhance low Pi tolerance of overexpression lines as *OsSPXs* are reported to operate as a negative regulator of Pi starvation responses in rice.

## Discussion

Root architecture has a major impact on the nutrient foraging capacity of plants (Morris et al. 2017). Agronomically, root length is one of the most important root traits particularly for immobile nutrients like Pi. Therefore, targeting mechanisms that regulate root length in crops have huge potential for alleviating nutrient stresses such as Pi deficiency.

Jasmonic acid is a key root growth inhibitory signal impacting root elongation (Staswick et al. 1992; Wang et al. 2002; Noir et al. 2013). To manipulate JA-mediated root growth inhibitory regulation, we employed JA signaling component (*OsJAZ11*) which is also low Pi inducible. *OsJAZ11* functions as a JA signaling repressor; hence, we anticipated its overexpression would suppress the root growth inhibitory effects of JA, potentially producing longer and more vigorous roots than WT which may enhance nutrient acquisition. Consistent with this model, *OsJAZ11* OE plants exhibited longer roots than WT under low Pi conditions (Fig. 3). Promotion of root length is a classical adaptive response of rice plants grown under low Pi (Shimizu et al. 2004). Surprisingly, *Arabidopsis* is reported to restrict primary root growth under low Pi conditions (Ticconi et al. 2004; Sánchez-Calderón et al. 2006). Moreover, *OsJAZ11* OE lines also displayed longer lateral and seminal roots than WT (Fig. 4), conferring an additional advantage to efficiently explore exogenous P resources. Consequently, P foraging capacity (total P uptake) of OE lines was significantly higher than WT under low Pi conditions (Fig. 2a). Overall, this led to enhanced biomass accumulation in *OsJAZ11* OE lines compared to WT, especially under low Pi stress.

**Fig. 7:**
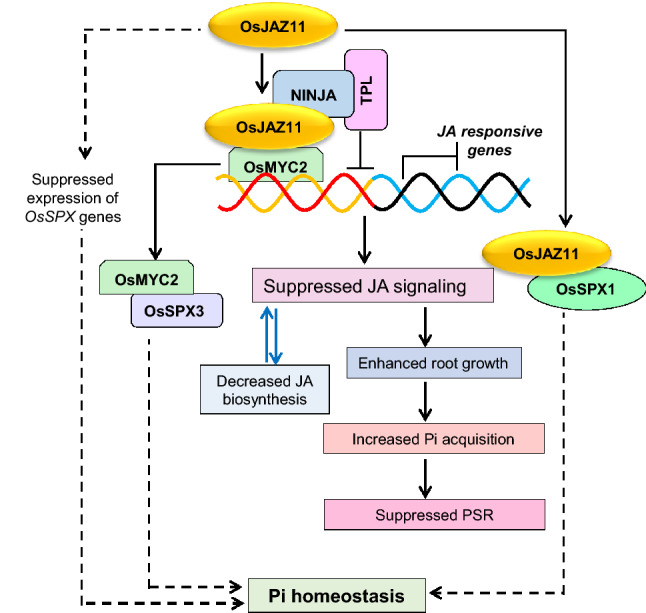
Schematic model illustrating mechanisms of action of *OsJAZ11* in rice. OsJAZ11 interacts with NINJA and TOPLESS (TPL) co-repressors. Upon its overexpression, OsJAZ11 blocks downstream transcription factors such as OsMYC2. Repressor activity of OsJAZ11 suppresses the expression of OsMYC2-regulated JA-responsive genes. This consequently increases root growth in overexpression lines. Enhanced root growth favors higher Pi acquisition and suppression of phosphate starvation response (PSR) in overexpression lines. *OsJAZ11* also regulates Pi homeostasis through *OsSPX*s. OsJAZ11 interacts with OsSPX1 and also alters expression of *OsSPX*s in overexpression lines under Pi deficiency. Other downstream component of *OsJAZ11* signaling pathway such as OsMYC2 interacts with key Pi homeostasis proteins such as OsSPX3 to regulate Pi homeostasis. All these mechanisms together support relatively enhanced plant growth in *OsJAZ11* overexpression lines under Pi deficiency

Till now, the molecular mechanisms through which JA and *OsJAZ11* participates in low Pi signaling have been unclear. Temporal expression analysis revealed that *OsJAZ11* is induced in low Pi conditions during early and late phases of low Pi stress (Supplementary Fig. S2). A previous study has also shown that *OsJAZ11* is strongly upregulated after 30 min of low nitrogen treatment in rice (Hsieh et al. 2018). This indicates important roles of OsJAZ11 in regulating early molecular events followed by nutrient stress signal. EMSA assays revealed that its low Pi induction is mediated by master regulator OsPHR2 (Fig. 1c). This suggests that *OsJAZ11* regulates low Pi signaling through an OsPHR2-dependent pathway. OsPHR2 is responsible for the induction of low-Pi-responsive genes during Pi stress (Liu et al. 2010; Mehra et al. 2017; Pandey et al. 2017). Notably, *OsJAZ11* overexpressing plants exhibited suppression of most of the low Pi marker genes at 30 days of Pi deficiency (Supplementary Fig. S5). To account the basis of this suppressed Phosphate Starvation Response (PSR), we propose two possibilities. First, *OsJAZ11* overexpressing plants accumulated relatively higher phosphate than WT owing to their improved root architecture. Higher Pi accumulation in OE lines might have suppressed PSR in OE lines leading to less induction of low-Pi-responsive marker genes as compared to WT. Alternatively, *OsJAZ11* might suppresses low Pi signaling by regulating (directly or indirectly) key genes in the low Pi signaling machinery. On the basis of improved root growth and direct interaction of OsJAZ11 with OsSPX1, we reason that *OsJAZ11* may exploit both strategies to maintain Pi homeostasis (Fig. 7). It is worth notable that we have determined the expression of Pi marker genes after 30 days of Pi deficiency. Nutrient deficiency responses are generally induced in a rhythmic manner. Therefore, plants may induce Pi deficiency responses when they perceive deficiency. After inducing these responses, they can get enough Pi and subsequently switch off the molecular responses. This implies that there might be variation in molecular (transcriptional/post-transcriptional) responses in WT versus transgenics during early and late Pi deficiency time-points.

Our study reports that OsJAZ11 physically interacts with the low Pi signaling component OsSPX1 and that most SPX family members are downregulated in *OsJAZ11* overexpressing plants under low Pi conditions (Fig. 6). SPX domains are regarded to function as low Pi sensors (Wild et al. 2016). The SPX domain binds to inositol pyrophosphates (PP-InsPs) with high selectivity and affinity (Wild et al. 2016). Cellular InsPs, especially InsP_8_ pools, are tightly regulated by nutrient availability (Dong et al. 2019). Under replete Pi conditions InsP_8_ accumulates, triggering formation of the SPX-InsP_8_−PHR complex (Dong et al. 2019). This complex inhibits binding of PHR to P1BS elements of low-Pi-responsive genes. Conversely, during Pi deficiency, InsP_8_ levels drop, leading to disassociation of the SPX-InsP_8_−PHR complex (Lorenzo-Orts et al. 2020). Consequently, PHR is released to induce low-Pi-responsive genes. Thus, SPX proteins acts as a negative regulator of PHR activity (Liu et al. 2010; Lv et al. 2014; Puga et al. 2014; Shi et al. 2014; Wang et al. 2014; Zhong et al. 2018). Interestingly, OsSPX1 is localized in the nucleus (Wang et al. 2014). Most of the JAZ proteins are also nuclear localized (Singh et al. 2020). A similar subcellular localization suggests functional relevance of OsJAZ11–OsSPX1 interaction. Our EMSA assay revealed that OsJAZ11–OsSPX1 interaction does not interfere with OsPHR2–OsSPX1-mediated regulation of PSR genes (Fig. 6c). This suggests that OsJAZ11 may regulate Pi homeostasis through OsSPX1 but independent of OsPHR2. The novel interaction of OsJAZ11 and SPX1 provides another mechanistic dimension to understand cross talk between low Pi and JA signaling pathways.

Functioning as a repressor of JA signaling, *OsJAZ11* overexpression is likely to suppress JA signaling through OsMYC2. We demonstrated that OsJAZ11 physically interacts with OsMYC2 and OsNINJA1. In addition, OsJAZ11 forms complexes with several TOPLESS co-repressors. These complexes can suppress *OsMYC2*-mediated JA signaling in OE lines. Moreover, *OsMYC2* and other JA-responsive *JAZ*s were downregulated in *OsJAZ11* OE lines (Supplementary Fig. S6). The reduced MeJA sensitivity of OE lines compared to WT further confirmed a reduced level of JA signaling in OsJAZ11 overexpressing lines (Supplementary Fig. S16). As a positive regulator of JA signaling, OsMYC2 controls expression of most of the JA-responsive genes (reviewed in Trang Nguyen et al. 2019). But it is unclear whether OsMYC2 directly participates in low P signaling. We examined the interaction of OsMYC2 with some of the key regulators of Pi signaling such as OsPHO1, OsPHO2, OsSIZ1, OsSPX1 and OsSPX3 (results not shown for all*)*. We found OsMYC2 interacts with OsSPX3 (Supplementary Fig. S20). These results again suggest that SPX domain containing proteins may function as common regulators of JA and Pi signaling.

Manipulating JA signaling also appears to perturb JA homeostasis in *OsJAZ11* OE lines. JA levels were significantly lower in *OsJAZ11* overexpressing roots (Supplementary Fig. S21). Strikingly, a low Pi regime caused even more of a decline in JA levels. Interestingly, lower levels of JA under Pi deficiency appear to be an adaptive response which may increase nutrient foraging by enhancing root growth. We also noticed decreased expression of JA biosynthetic genes like *OsAOS2* in *OsJAZ11* overexpressing roots. Hence, *OsJAZ11* overexpression leads to reduced JA signaling as well JA levels. It is interesting to note that contrary to classical low phosphate responses, Fe deficiency promotes higher uptake of phosphate in rice and concomitantly low Pi machinery is suppressed (Zheng et al. 2009). Moreover, unlike P deficiency, Fe deficiency reduces rice root length (Sun et al 2017). Given this contrary behavior, we found that unlike the enhanced JA response during early Fe deficiency (Kobayashi et al. 2016), both, JA level and response, were suppressed in our low P conditions in rice root.

To date, it has been unclear why rice and *Arabidopsis* exhibit contrasting root adaptive responses to low P conditions. *Arabidopsis* suppresses primary root growth which results in elongation of lateral branches. This adaptive response (excessive branching/shallow root growth) is believed to provide greater access to sparsely distributed phosphate accumulating in top soil. In contrast, rice plants have a fibrous root system where enhanced length of its seminal/adventitious roots aids foraging the maximum soil volume. What is the basis for these contrasting growth responses? In our present study, we observed a decreased accumulation of JA in rice roots under Pi deficient conditions, which would stimulate root elongation (Supplementary Figs. S21, S22). A recent report revealed that low Pi results in higher JA accumulation in *Arabidopsis* roots, which would serve to inhibit root elongation (Khan et al. 2016). To validate this latter observation, we employed the *Arabidopsis* JA biosensor JAS9:VENUS (Larrieu et al. 2015). This biosensor revealed higher JA accumulation in Arabidopsis primary roots under low Pi conditions since almost no VENUS signal was detected in these roots when compared with the +P ones (Supplementary Fig. S23). These contrasting JA responses help to explain the contrasting root growth phenotypes in rice and *Arabidopsis* under Pi deficiency.

In summary, our study not only provides novel resources and mechanistic insights to improve Pi utilization ability in rice, but also reveals a novel role for JA signaling in improving low Pi tolerance of crop plants. The JA signaling mechanisms described in our present study open new avenues to alleviate Pi deficiency in other cereal crops through genetic engineering.

### *Author contribution statement*

PM, JG and BKP conceived the original research plans. BKP generated all the transgenic resources. PM designed and conducted most of the experiments. PM, JG and BKP analyzed the data. LV and APS participated in plant phenotyping and P quantitation. AP contributed in Y2H and RT-qPCR assays. JG, AKT and MJB discussed research plans and outcomes. JG, AKT and MJB edited the manuscript. PM and BKP wrote the article. All authors read, edited and approved the final version of the manuscript.

## Supplementary Information

Below is the link to the electronic supplementary material.

Supplementary file1 (PDF 2898 KB) Table S1 List of primers used in the study. Table S2 List of interacting proteins identified in OsJAZ11 OE lines using MS using Exactive™ Plus Orbitrap Mass Spectrometer. Supplementary Fig. S1 OsJAZ11 is a JA-responsive gene. a Schematic representation of gene model of OsJAZ11 (1551 bp) and open reading frame (ORF) (209 a.a.). Positions of intron/exons in gene structure (above panel) and motifs in ORF (below panel) have been indicated. b Expression profile of OsJAZ11 retrieved from microarray database RiceXPro (Rice Expression Profile Database) version 3.0. Error bars represent standard deviation (n=3). c Histochemical localization of GUS signals in coleoptiles of pOsJAZ11:GUS rice transgenics subjected to different doses of MeJA treatments (0, 1, 10, 50 and 100 μM) for 1 h. Supplementary Fig. S2 OsJAZ11 is induced by local and systemic Pi fluctuations. a-b Relative expression profiles of OsJAZ11 and OsIPS1 in roots of PB1 seedlings. Seven-day-old rice seedlings grown under +P conditions were transferred to −P conditions. OsIPS1 was used as a marker gene to depict onset of Pi deficiency response at molecular levels. Gene expression levels were measured at indicated time points by RT-qPCR and fold changes were determined with respect to 0 time point. c Soluble Pi content in rice seedlings at time points corresponding to time points in panel a and b. Each bar represents mean of three independent replicates with standard error (n=3). Supplementary Fig. S3 Raising of OsJAZ11 transgenics. a Schematic representation of OsJAZ11 overexpression construct in Gateway-compatible destination vector, pANIC6B. OsJAZ11 was overexpressed under maize Ubiquitin promoter (ZmUbi1). b Schematic representation of OsJAZ11 silencing construct in Gateway-compatible destination vector, pANIC8B. ZmUbi1 (maize ubiquitin 1 promoter and intron), R1 and R2 (attR1 and attR2 recombination sites), AcV5 (epitope tag), OCS T (octopine synthase terminator sequence). c-d Relative expression levels of OsJAZ11 in roots of thirty-day-old OsJAZ11 overexpression (OE) and RNAi (Ri) transgenics compared to WT. Each bar represents mean of three biological replicates with standard error. Significant differences between WT and transgenics were evaluated by Student’s *t* test. Asterisks; * and *** indicate P values, ≤ 0.05 and 0.001, respectively (n=3). Supplementary Fig. S4 OsJAZ11 OE lines showed higher phosphorous uptake. a-b P content of roots of thirty-day-old plants of WT and OsJAZ11 transgenics under +P and −P conditions. c P content of shoots of thirty-day-old plants of WT and OsJAZ11 transgenics under +P conditions. Each bar displays means of ten biological replicates with standard error. Significant differences between WT and transgenics were determined by Student’s *t* test. Asterisks; *, ** and *** indicate P values, ≤ 0.05, 0.01 and 0.001, respectively (n=10). Supplementary Fig. S5 OsJAZ11 OE lines displayed suppressed phosphate starvation response (PSR). a–i Relative expression levels of low Pi marker genes OsPT1, OsPT4, OsPT8, OsPT9, OsIPS1, OsGDPD5, OsMGD3, OsPAP3b and OsPAP10a. Expression levels were measured by RT-qPCR in roots of thirty-day-old WT and OsJAZ11 transgenics under Pi deficiency. Each bar displays means of three biological replicates with standard error. Significant differences between WT and transgenics were determined by Student’s *t* test. Asterisks; *, ** and *** indicate P values, ≤ 0.05, 0.01 and 0.001, respectively (n=3). Supplementary Fig. S6 Overexpression of OsJAZ11 suppressed expression of JA biosynthesis and signaling genes. a–g Relative expression levels of OsJAZ4, OsJAZ8, OsJAZ9, OsAOS1, OsAOS2, OsOPR1 and OsMYC2. Expression levels were measured by RT-qPCR in roots of thirty-day-old WT and OsJAZ11 transgenics under Pi deficiency. Each bar displays means of three biological replicates with standard error. Significant differences between WT and transgenics were determined by Student’s *t* test. Asterisks; *, ** and *** indicate P values, ≤ 0.05, 0.01 and 0.001, respectively (n=3). Supplementary Fig. S7 Root length of fifteen-day-old WT and OsJAZ11 transgenics. Root phenotype of fifteen-day-old OsJAZ11 overexpression (OE) lines (a, d) and silencing RNAi (Ri) lines (b, e) compared to WT under +P (a, b) and −P (d, e) conditions. For imaging three representative plants of each line were stacked together. Scale bar = 10 cm. c Quantitation of root lengths of WT, OE and Ri lines under +P conditions. f Quantitation of root lengths of WT, OE and Ri lines under −P conditions. Each bar represents mean of ten biological replicates with standard error. Significant differences between WT and transgenics were determined by Student’s *t* test. Asterisks; *, ** and *** indicate P values, ≤ 0.05, 0.01 and 0.001, respectively (n=10). Ns denotes no significant differences between WT and transgenics. Supplementary Fig. S8 OsJAZ11 overexpression lines accumulated higher biomass under Pi deficiency. a-b Root and shoot dry biomass of thirty-day-old WT and OsJAZ11 OE and RNAi (Ri) transgenics under +P and −P conditions. Each bar depicts mean of ten biological replicates with standard error. Significant differences between WT and transgenics were determined by Student’s *t* test. Asterisks; *, ** and *** indicate P values, ≤ 0.05, 0.01 and 0.001, respectively (n=10). Supplementary Fig. S9 Effect of OsJAZ11 on different root traits. Seminal root number (a), lateral root length per cm of root (b) and lateral root density (number of laterals per cm of main root) (c). Root traits were measured in roots of fifteen-day-old WT and OsJAZ11 transgenics under +P and −P conditions. Each bar represents mean of four replicates with standard error (n=4). Supplementary Fig. S10 OsJAZ11 shows in vivo repressor activity. a Raising of OsJAZ11 translational reporters. Schematic representation of OsJAZ11-GUS (with Jas motif) and OsJAZ11ΔC-GUS (without Jas motif) fusion constructs in pCAMBIA1301. In OsJAZ11ΔC-GUS, 57 a.a. (153- 209 a.a.) from C-terminal (CT) end of OsJAZ11 ORF was deleted. This deleted region also contains Jas motif. b Relative expression levels of OsJAZ11 in WT, OsJAZ11-GUS (L12, L14) and OsJAZ11ΔC-GUS (L1, L2) transgenics. Expression levels were measured by RT-qPCR in roots of thirty-day-old plants. Each bar represents mean of three biological replicates with standard error. Significant differences between WT and transgenics were determined by Student’s *t* test. Asterisks; *** indicate P value, ≤ 0.001 (n=3). c–d Fluorometric quantitation of GUS signals in roots of fifteen-day-old OsJAZ11-GUS (L14) and OsJAZ11ΔC-GUS (L1) transgenics subjected to MeJA and/or MG132 treatments. Plants were treated with 100 μM MeJA with or without the proteasome inhibitor 100 μM MG132 for 1 h. DMSO treated seedlings were used as control. Each bar represents mean of four biological replicates with standard error (n=4). e–f Representative images showing GUS signals in root tips of fifteen-day-old OsJAZ11-GUS (L14) and OsJAZ11ΔC-GUS (L1) transgenics treated MeJA and/or MG132 treatments. Supplementary Fig. S11 Root length of fifteen-day-old WT and OsJAZ11 translational reporters. Root phenotype of fifteen-day-old OsJAZ11-GUS (a, d) and OsJAZ11ΔC-GUS lines (b, e) compared to WT under +P (a, b) and −P (d, e) conditions. For imaging three representative plants of each line were stacked together. Scale bar = 10 cm. c Quantitation of root lengths of WT and translational reporters under +P conditions. f Quantitation of root lengths of WT and translational reporters under −P conditions. Each bar represents mean of ten biological replicates with standard error. Significant differences between WT and transgenics were determined by Student’s *t* test. Asterisks; ** and *** indicate P values, ≤ 0.01 and 0.001, respectively. Ns denotes no significant differences between WT and transgenics (n=10). Supplementary Fig. S12 OsJAZ11-GUS lines developed longer seminal roots. Average seminal root length (a) and total seminal root length (b) of fifteen-day-old WT, OsJAZ11-GUS (L12, L14) and OsJAZ11ΔC-GUS (L1, L2) lines under +P and −P conditions. Each bar represents mean from four replicates with standard error. Significant differences between WT and transgenics were determined by Student’s *t* test. Asterisks; * and ** indicate P values, ≤ 0.05 and 0.01, respectively (n=4). c Representative images of fifteen-day-old WT and OsJAZ11 translational reporters under +P and −P conditions. White line at bottom of each image denotes scale of 1 cm. Supplementary Fig. S13 OsJAZ11-GUS lines accumulated more biomass under Pi deficiency. Root and shoot dry biomass of thirty-day-old WT, OsJAZ11-GUS (L12, L14) and OsJAZ11ΔC-GUS (L1, L2) lines under +P (a) and −P (b) conditions. Each bar represents mean of ten biological replicates with standard error. Significant differences between WT and transgenics were determined by Student’s *t* test. Asterisks; *, ** and *** indicate P values, ≤ 0.05, 0.01 and 0.001, respectively (n=10). Supplementary Fig. S14 OsJAZ11-GUS transgenics accumulated higher Pi than OsJAZ11ΔC-GUS transgenics. Seven-day-old rice seedlings of OsJAZ11-GUS (L12, L14) and OsJAZ11ΔC-GUS (L1, L2) lines grown under +P conditions were transferred to –P conditions. Soluble Pi was measured at indicated time-points. Means were calculated from three biological replicates. Error bars indicate standard error (n=3). Supplementary Fig. S15 OsJAZ11-GUS lines showed higher phosphorous uptake under Pi deficiency. a–b P content of roots of thirty-day-old plants of WT, OsJAZ11-GUS (L12, L14) and OsJAZ11ΔC-GUS (L1, L2) lines under +P and −P conditions. c-d P content of shoots of thirty-day-old plants of WT, OsJAZ11-GUS (L12, L14) and OsJAZ11ΔC-GUS (L1, L2) lines under +P and −P conditions. Each bar displays means of ten biological replicates with standard error. Significant differences between WT and transgenics were determined by Student’s *t* test. Asterisks; *, ** and *** indicate P values, ≤ 0.05, 0.01 and 0.001, respectively (n=10). Supplementary Fig. S16 JA sensitivity assay of OsJAZ11 transgenics. a Root images of WT and OsJAZ11 transgenics subjected to DMSO (control) and MeJA (10 μM) treatment. Seven-day-old rice seedlings were treated with 10 μM MeJA or DMSO (control) for 14 days. For imaging three representative plants of each line were stacked together. Scale bar = 5 cm. b Percent reduction in root lengths of WT and OsJAZ11 transgenics after MeJA treatment. Reduction in root length under MeJA treatment was compared to control conditions (n=10). Significant differences between WT and transgenics were determined by Student’s *t* test. Asterisks; *, ** and *** indicate P values, ≤ 0.05, 0.01 and 0.001, respectively (n=10). Supplementary Fig. S17 Interactions assays of OsJAZ11 with JA and Pi signaling proteins. Yeast two-hybrid interaction assays between bait plasmid, pBD-OsJAZ11 and prey plasmids, pAD-OsMYC2, pAD-OsNINJA1, pAD-OsPHO2, pAD-OsSPX3, pAD-OsPHO1.2. Serial dilutions of Y2H Gold strain co-transformed with AD (prey) and BD (bait) clones were spotted on DDO (SD-Leu/-Trp), TDO (SD- Leu/-Trp/-His), QDO (SD- Leu/-Trp/-His/-Ade) and QDO/A/X (QDO + Aureobasidin A + X-α-Gal). pBD and pAD indicates empty BD (pGBKT7) and AD (pGADT7) vectors, respectively. Interaction between pAD-T-Antigen and pBD-p53 was used as positive control whereas interaction between pAD-T-Antigen and pBD-Lam was used as a negative control. Supplementary Fig. S18 Induction and purification of OsJAZ11-GST. a Overexpression of OsJAZ11-GST in E. coli BL21(DE3) induced by IPTG. Induced (IN) and uninduced (UN) total protein fractions were resolved on 12% SDS−PAGE gel. Gel was stained with Coomassie blue. P and S implies proteins in pellet (insoluble) and supernatant (soluble) fractions. 20 μg of total protein was loaded in each lane. b Coomassie-stained SDS−PAGE gel showing 49.9 kDa purified recombinant OsJAZ11-GST protein. Supplementary Fig. S19 Expression of OsSPX4 in WT and OsJAZ11 transgenics. Relative expression levels of OsSPX4 in roots of thirty-day-old WT and OsJAZ11 transgenics under +P and −P conditions. Each bar is the mean of three independent replicates with standard error. Different letters on top of each bar denotes significant differences determined by one-way ANOVA followed by Duncan’s multiple comparison test (α < 0.05) (n=3). Supplementary Fig. S20 Interactions assays of OsMYC2 with SPX proteins. Yeast two-hybrid interaction assays between pAD-OsMYC2 and pBD-OsSPX (OsSPX1 and OsSPX3). Cells of yeast strain, Y2H Gold co-transformed with AD (prey) and BD (bait) clones were spotted on DDO (SD-Leu/-Trp), TDO (SD- Leu/-Trp/-His) and QDO (SD- Leu/-Trp/-His/-Ade). pBD and pAD indicates empty BD (pGBKT7) and AD (pGADT7) vectors, respectively. Interaction between pAD-T-Antigen and pBD-p53 was used as positive control, whereas interaction between pAD-T-Antigen and pBD-Lam was used as a negative control. Supplementary Fig. S21 Pi deficiency reduces JA levels in rice. JA content in roots of thirty-day-old WT and OsJAZ11 OE transgenics grown under +P and −P conditions. Each bar represents mean of six independent replicates with standard error. Significant differences between WT and transgenics were evaluated by Student’s *t* test (n=6). Supplementary Fig. S22 JA levels in rice under progressive Pi deficiency. JA levels (a) and soluble Pi content (b) in roots of PB1 seedlings. Seven-day-old rice seedlings grown under +P conditions were transferred to –P conditions. Soluble Pi and JA levels were measured at indicated time-points. For JA and Pi measurements means were calculated from six (n=6) and four (n=4) biological replicates, respectively. Error bars indicate standard error. Different letters on top of each bar denotes significant differences determined by one-way ANOVA followed by Duncan’s multiple comparison test (α < 0.05). Supplementary Fig. S23 Pi deficiency leads to root length inhibition and increased JA levels in Arabidopsis. a Plant phenotype of ten-day-old Arabidopsis seedlings (Col-0) grown under Pi sufficient (HP) and deficient (LP) conditions. Scale bar =1 cm. b Quantitation of primary root length of Arabidopsis seedling grown under Pi sufficient (HP) and deficient (LP) conditions. Each bar represent mean of ten replicates with standard error. c Representative SP5 confocal images showing Jas9-VENUS fluorescence in primary root tips of Arabidopsis under HP and LP conditions. d Quantitation of VENUS fluorescence under HP and LP conditions. Significant differences between HP and LP conditions were determined by Student’s *t* test. Asterisks; *** indicate P value ≤ 0.001 (n=10).

## Data Availability

All data generated or analyzed during this study are included in this published article and its supplementary information files.

## Abbreviations

JA: Jasmonic acid
JAZ: JASMONATE ZIM-DOMAIN
OE: Overexpression
PHR: Phosphate starvation response regulator
P1BS: PHR1 binding sequence
Pi: Inorganic phosphate
PSR: Pi starvation response
