# Supplementary material for: OsJAZ11 regulates phosphate starvation responses in rice
Source: Planta. 2021 Jun 18;254(1):8. doi: 10.1007/s00425-021-03657-6 (PMC8213676; doi:10.1007/s00425-021-03657-6)
Supplement: Supplementary file 1 — Supplementary file1 (PDF 2898 KB) Table S1 List of primers used in the study. Table S2 List of interacting proteins identified in OsJAZ11 OE lines using MS using Exactive™ Plus Orbitrap Mass Spectrometer. Supplementary Fig. S1 OsJAZ11 is a JA-responsive gene. a Schematic representation of gene model of OsJAZ11 (1551 bp) and open reading frame (ORF) (209 a.a.). Positions of intron/exons in gene structure (above panel) and motifs in ORF (below panel) have been indicated. b Expression profile of OsJAZ11 retrieved from microarray database RiceXPro (Rice Expression Profile Database) version 3.0. Error bars represent standard deviation (n=3). c Histochemical localization of GUS signals in coleoptiles of pOsJAZ11:GUS rice transgenics subjected to different doses of MeJA treatments (0, 1, 10, 50 and 100 μM) for 1 h. Supplementary Fig. S2 OsJAZ11 is induced by local and systemic Pi fluctuations. a-b Relative expression profiles of OsJAZ11 and OsIPS1 in roots of PB1 seedlings. Seven-day-old rice seedlings grown under +P conditions were transferred to −P conditions. OsIPS1 was used as a marker gene to depict onset of Pi deficiency response at molecular levels. Gene expression levels were measured at indicated time points by RT-qPCR and fold changes were determined with respect to 0 time point. c Soluble Pi content in rice seedlings at time points corresponding to time points in panel a and b. Each bar represents mean of three independent replicates with standard error (n=3). Supplementary Fig. S3 Raising of OsJAZ11 transgenics. a Schematic representation of OsJAZ11 overexpression construct in Gateway-compatible destination vector, pANIC6B. OsJAZ11 was overexpressed under maize Ubiquitin promoter (ZmUbi1). b Schematic representation of OsJAZ11 silencing construct in Gateway-compatible destination vector, pANIC8B. ZmUbi1 (maize ubiquitin 1 promoter and intron), R1 and R2 (attR1 and attR2 recombination sites), AcV5 (epitope tag), OCS T (octopine synthase terminator sequence). c-d [file 425_2021_3657_MOESM1_ESM.pdf]

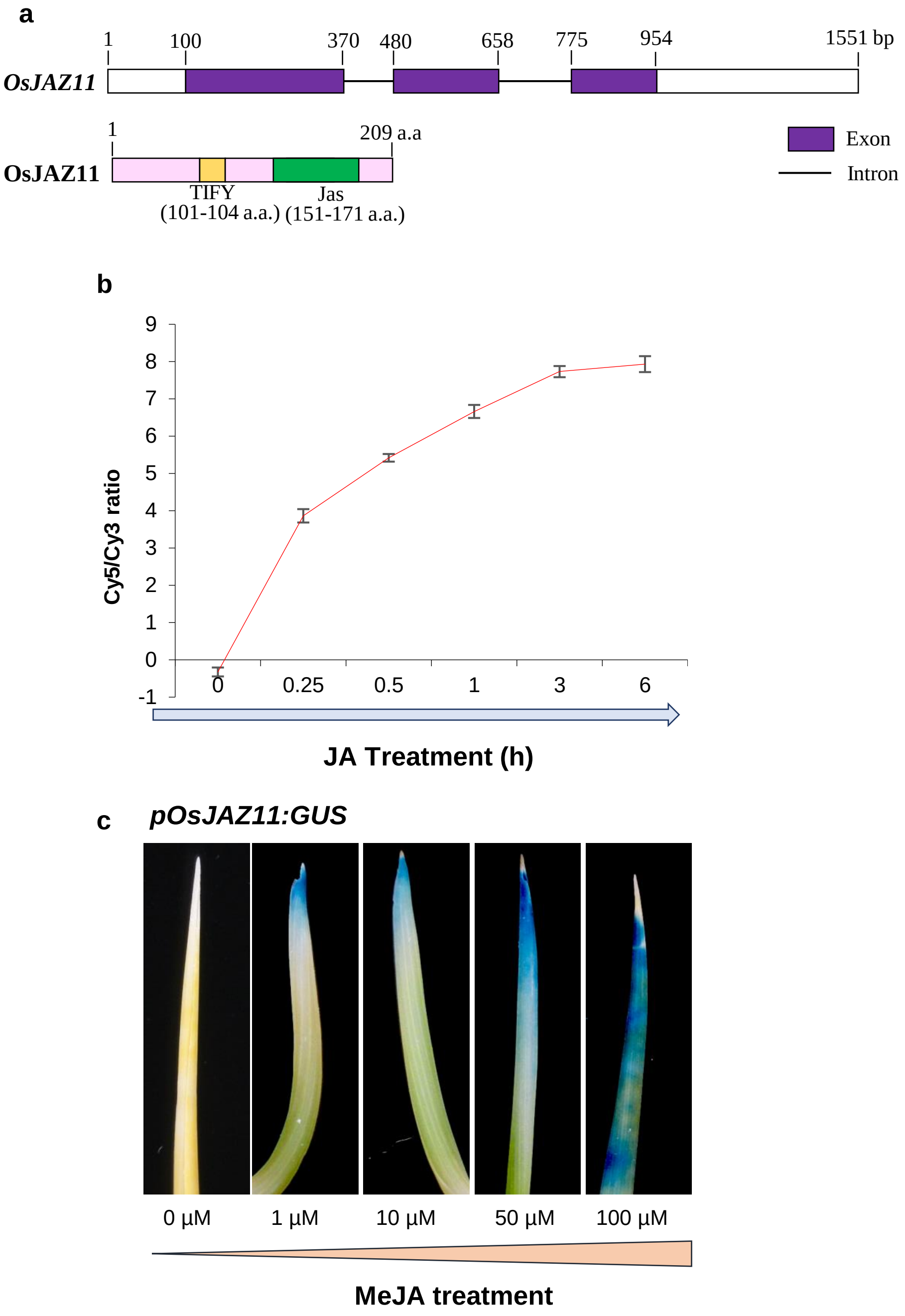

Supplementary Fig. S1 *OsJAZ11* is a JA responsive gene. **a** Schematic representation of gene model of *OsJAZ11* (1551 bp) and open reading frame (ORF) (209 a.a.). Positions of intron/exons in gene structure (above panel) and motifs in ORF (below panel) have been indicated. **b** Expression profile of *OsJAZ11* retrieved from microarray database RiceXPro (Rice Expression Profile Database) version 3.0. Error bars represent standard deviation (n=3). **c** Histochemical localization of GUS signals in coleoptiles of *pOsJAZ11:GUS* rice transgenics subjected to different doses of MeJA treatments (0, 1, 10, 50 and 100 µM) for 1 h.

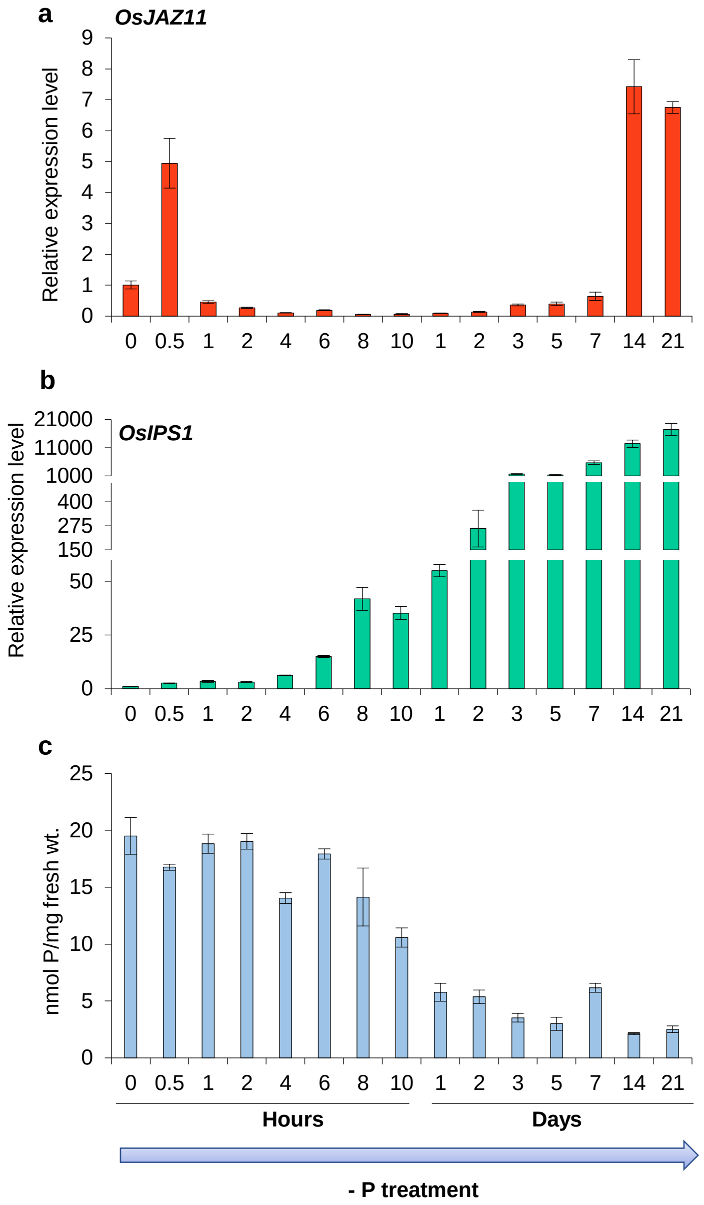

Supplementary Fig. S2 *OsJAZ11* is induced by local and systemic Pi fluctuations. **a-b** Relative expression profiles of *OsJAZ11* and *OsIPS1* in roots of PB1 seedlings. Seven-day-old rice seedlings grown under +P conditions were transferred to -P conditions. *OsIPS1* was used as a marker gene to depict onset of Pi deficiency response at molecular levels. Gene expression levels were measured at indicated time points by RT-qPCR and fold changes were determined with respect to 0 time point. **c** Soluble Pi content in rice seedlings at time points corresponding to time points in panel **a** and **b**. Each bar represents mean of three independent replicates with standard error (*n*=3).

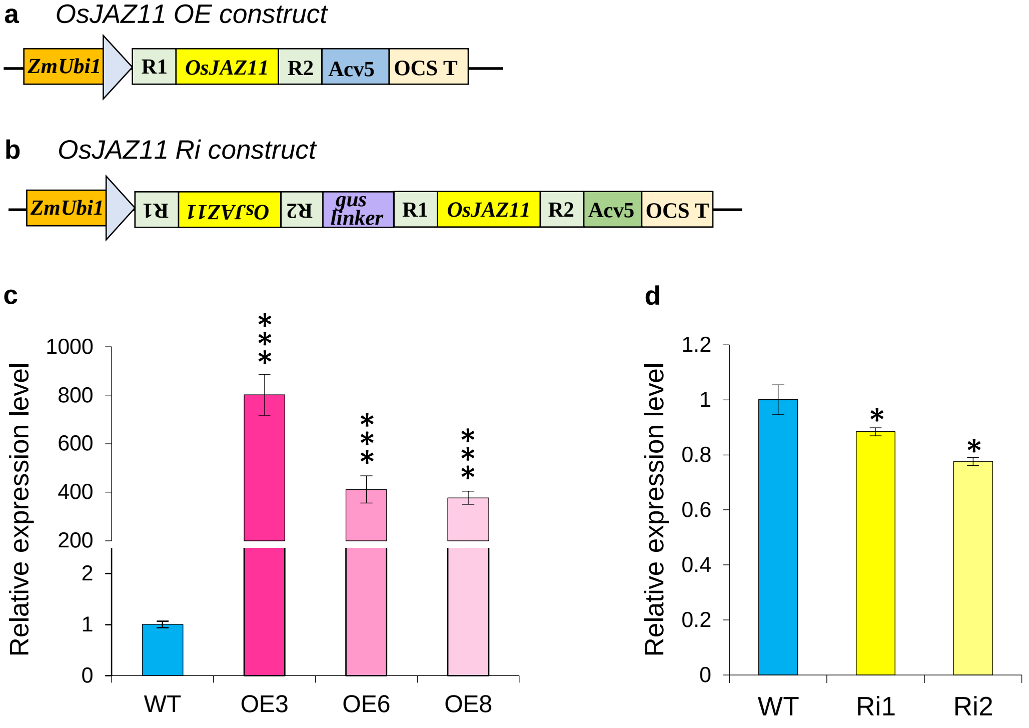

Supplementary Fig. S3 Raising of *OsJAZ11* transgenics. **a** Schematic representation of *OsJAZ11* overexpression construct in Gateway-compatible destination vector, pANIC6B. *OsJAZ11* was overexpressed under maize *Ubiquitin* promoter (*ZmUbi1*). **b** Schematic representation of *OsJAZ11* silencing construct in Gateway-compatible destination vector, pANIC8B. *ZmUbi1* (maize ubiquitin 1 promoter and intron), R1 and R2 (*attR1* and *attR2* recombination sites), AcV5 (epitope tag), *OCS* T (octopine synthase terminator sequence). **c-d** Relative expression levels of *OsJAZ11* in roots of thirty-day-old *OsJAZ11* overexpression (OE) and RNAi (Ri) transgenics compared to WT. Each bar represents mean of three biological replicates with standard error. Significant differences between WT and transgenics were evaluated by Student's *t*-test. Asterisks; * and *** indicate *P* values, ≤ 0.05 and 0.001, respectively (*n*=3).

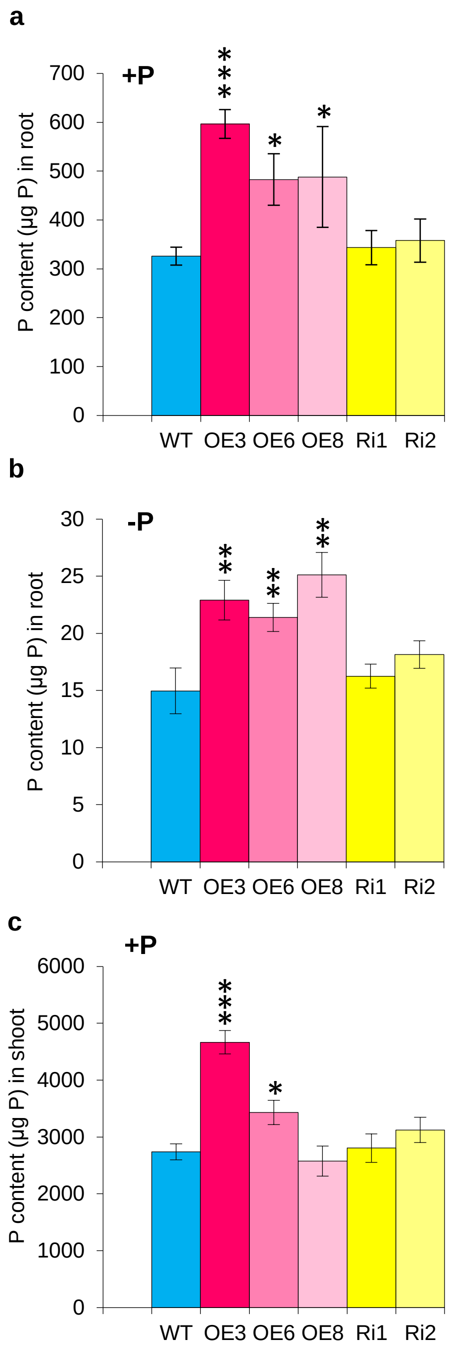

Supplementary Fig. S4 *OsJAZ11* OE lines showed higher phosphorous uptake. **a-b** P content of roots of thirty-day-old plants of WT and *OsJAZ11* transgenics under +P and -P conditions. **c** P content of shoots of thirty-day-old plants of WT and *OsJAZ11* transgenics under +P conditions. Each bar displays means of ten biological replicates with standard error. Significant differences between WT and transgenics were determined by Student's *t*-test. Asterisks; *, ** and *** indicate *P* values, ≤ 0.05, 0.01 and 0.001, respectively (*n*=10).

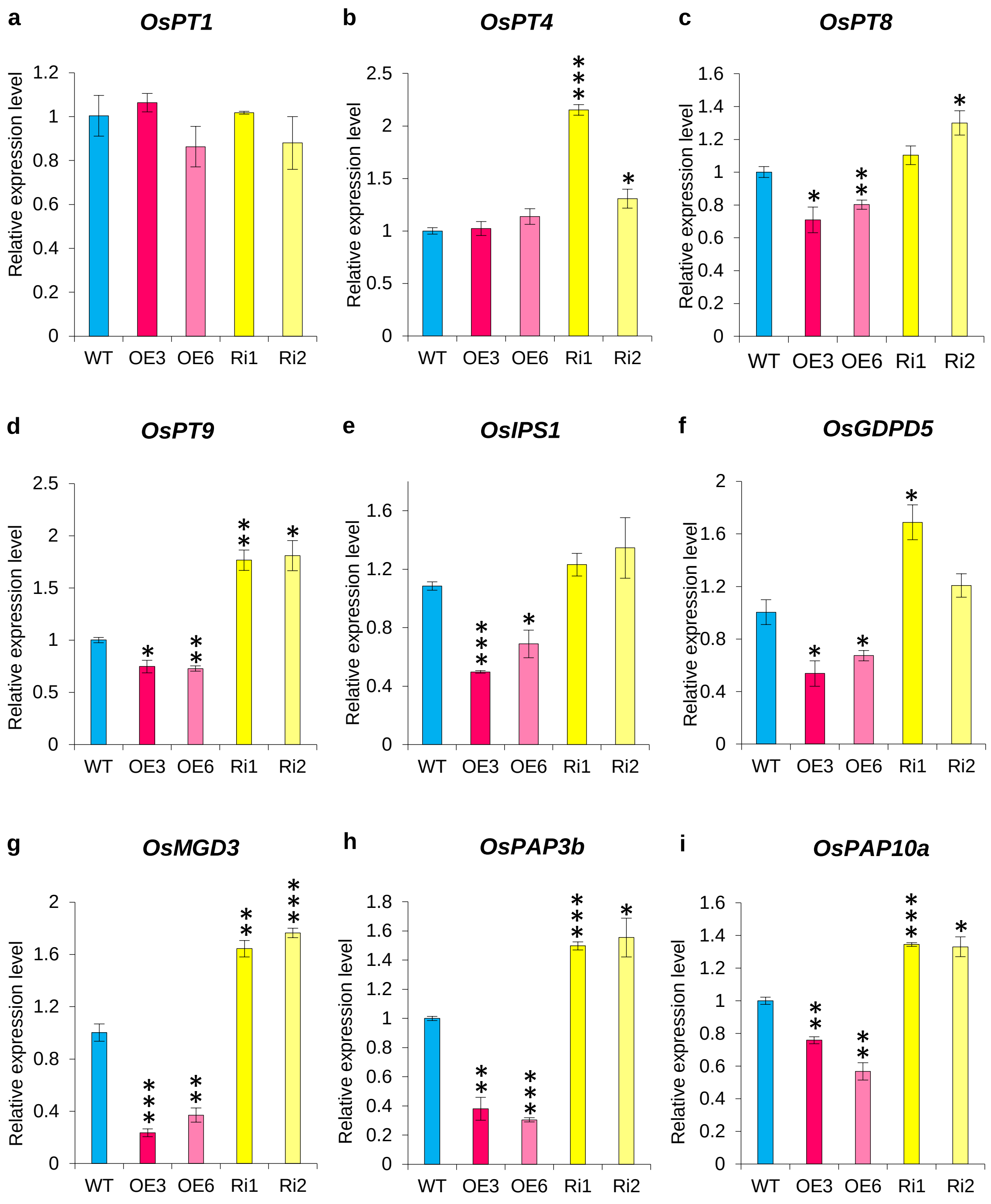

Supplementary Fig. S5 *OsJAZ11* OE lines displayed suppressed phosphate starvation response (PSR). **a-i** Relative expression levels of low Pi marker genes *OsPT1*, *OsPT4*, *OsPT8*, *OsPT9*, *OsIPS1*, *OsGDPD5*, *OsMGD3*, *OsPAP3b* and *OsPAP10a*. Expression levels were measured by RT-qPCR in roots of thirty-day-old WT and *OsJAZ11* transgenics under Pi deficiency. Each bar displays means of three biological replicates with standard error. Significant differences between WT and transgenics were determined by Student's *t*-test. Asterisks; *, ** and *** indicate *P* values, ≤ 0.05, 0.01 and 0.001, respectively (*n*=3).

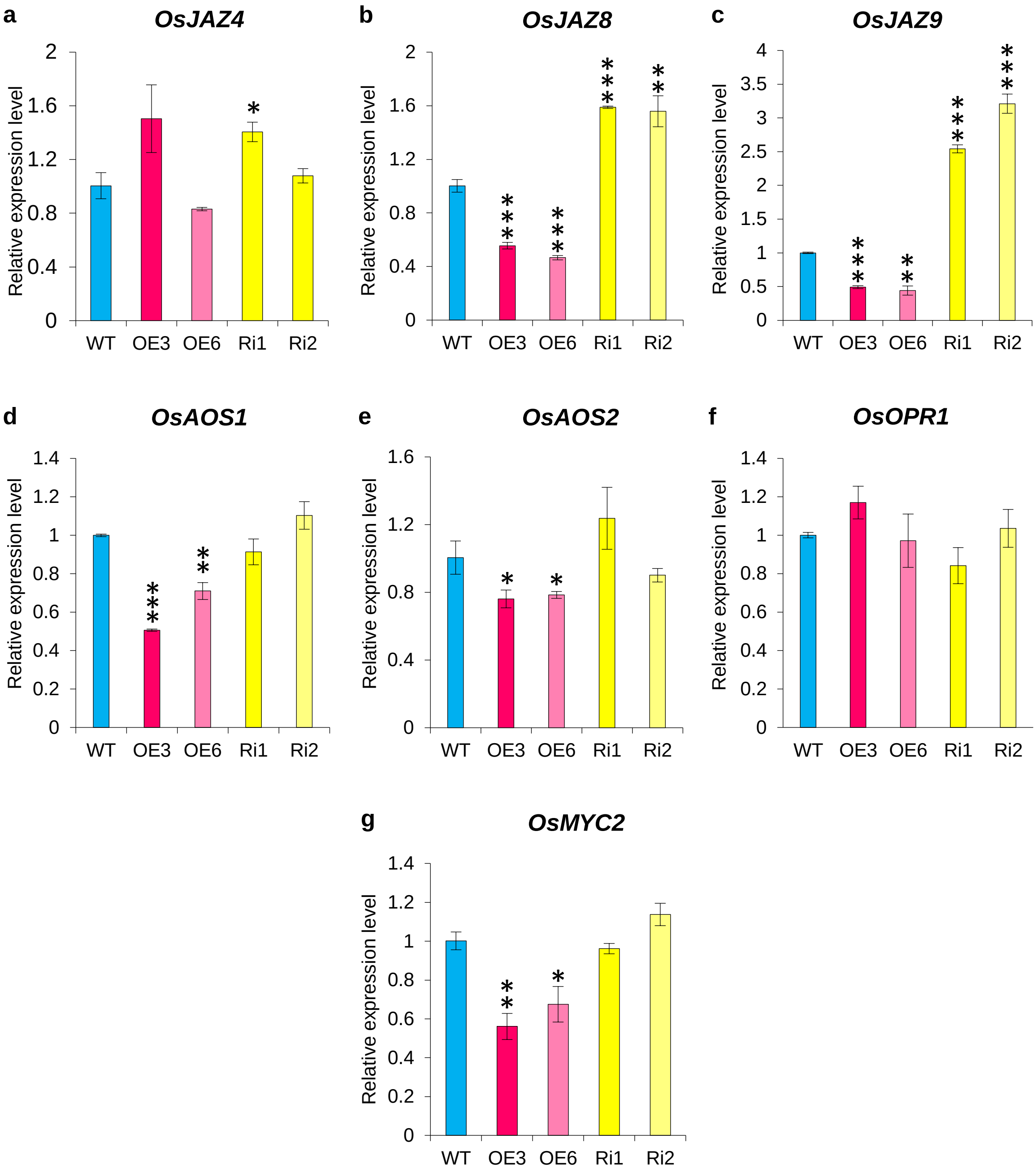

Supplementary Fig. S6 Overexpression of *OsJAZ11* suppressed expression of JA biosynthesis and signalling genes. **a-g** Relative expression levels of *OsJAZ4*, *OsJAZ8*, *OsJAZ9*, *OsAOS1*, *OsAOS2*, *OsOPR1* and *OsMYC2*. Expression levels were measured by RT-qPCR in roots of thirty-day-old WT and *OsJAZ11* transgenics under Pi deficiency. Each bar displays means of three biological replicates with standard error. Significant differences between WT and transgenics were determined by Student's *t*-test. Asterisks; *, ** and *** indicate *P* values, ≤ 0.05, 0.01 and 0.001, respectively (*n*=3).

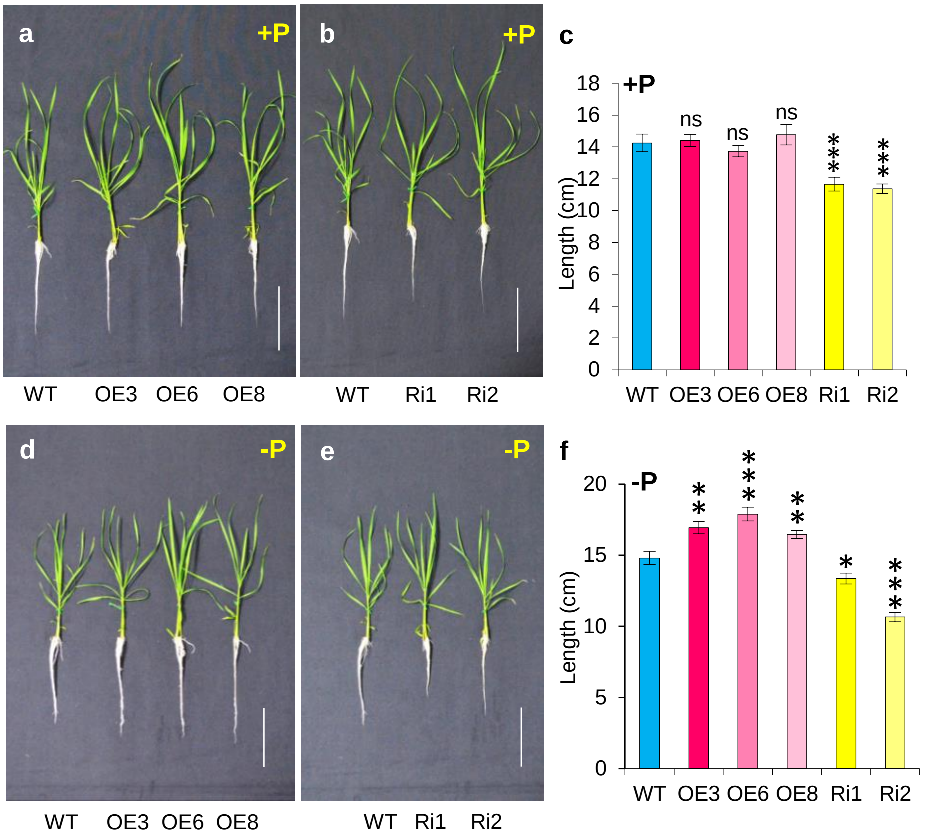

Supplementary Fig. S7 Root length of fifteen-day-old WT and *OsJAZ11* transgenics. Root phenotype of fifteen-day-old *OsJAZ11* overexpression (OE) lines (**a**, **d**) and silencing (Ri) lines (**b**, **e**) compared to WT under +P (**a**, **b**) and -P (**d**, **e**) conditions. For imaging three representative plants of each line were stacked together. Scale bar = 10 cm. **c** Quantitation of root lengths of WT, OE and Ri lines under +P conditions. **f** Quantitation of root lengths of WT, OE and Ri lines under -P conditions. Each bar represents mean of ten biological replicates with standard error. Significant differences between WT and transgenics were determined by Student's *t*-test. Asterisks; *, ** and *** indicate *P* values, ≤ 0.05, 0.01 and 0.001, respectively (*n*=10). Ns denotes no significant differences between WT and transgenics.

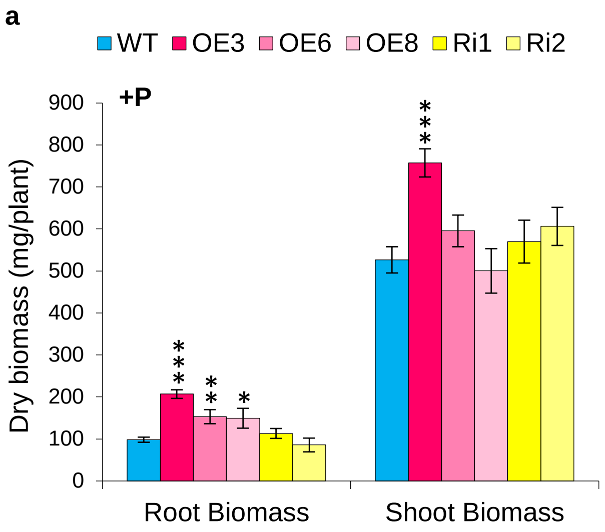

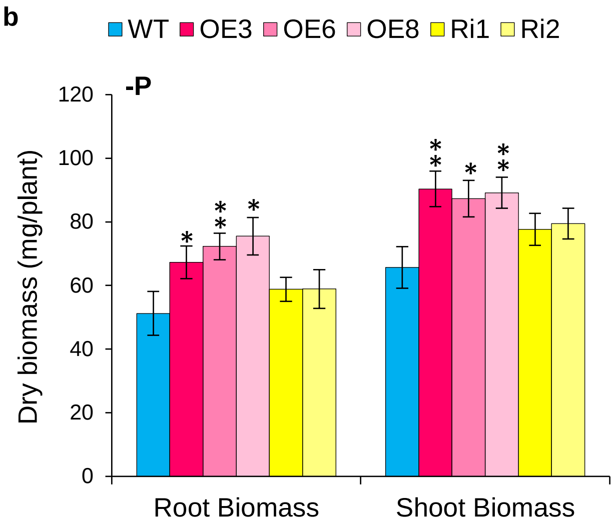

Supplementary Fig. S8 *OsJAZ11* overexpression lines accumulated higher biomass under Pi deficiency. **a-b** Root and shoot dry biomass of thirty-day-old WT and *OsJAZ11* OE and Ri transgenics under +P and -P conditions. Each bar depicts mean of ten biological replicates with standard error. Significant differences between WT and transgenics were determined by Student's *t*-test. Asterisks; *, ** and *** indicate *P* values, ≤ 0.05, 0.01 and 0.001, respectively (*n*=10).

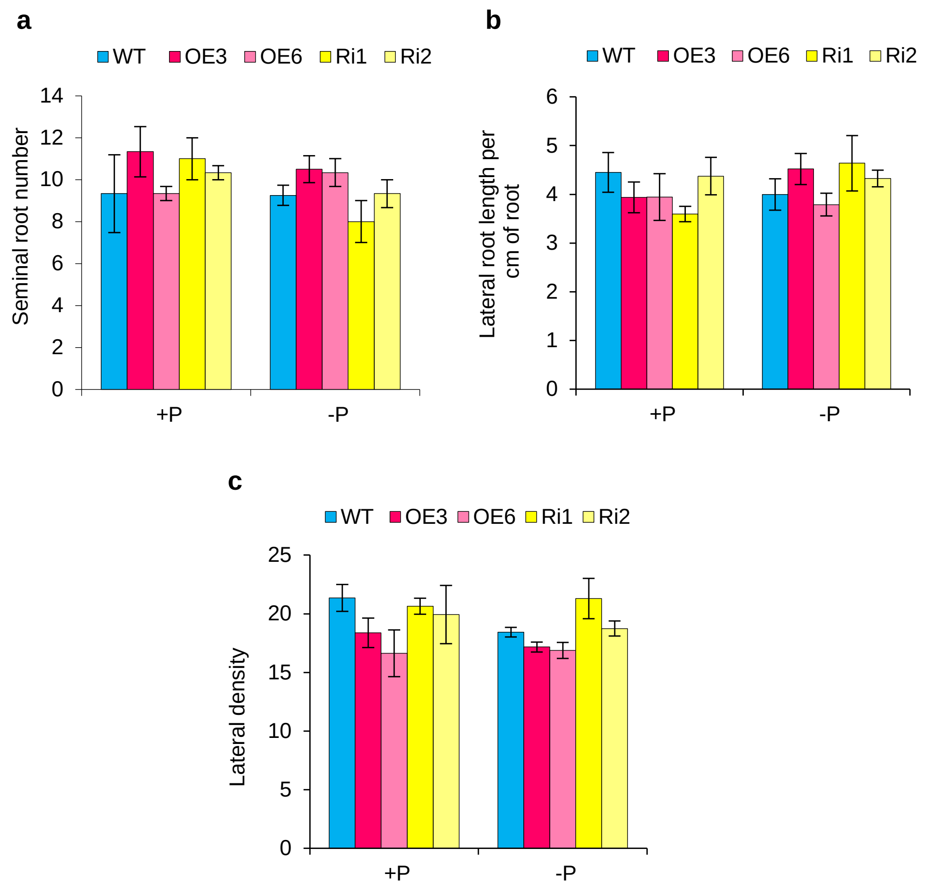

Supplementary Fig. S9 Effect of *OsJAZ11* on different root traits. Seminal root number (**a**), lateral root length per cm of root (**b**) and lateral root density (number of laterals per cm of main root) (**c**). Root traits were measured in roots of fifteen-day-old WT and *OsJAZ11* transgenics under +P and -P conditions. Each bar represents mean of four replicates with standard error (*n*=4).

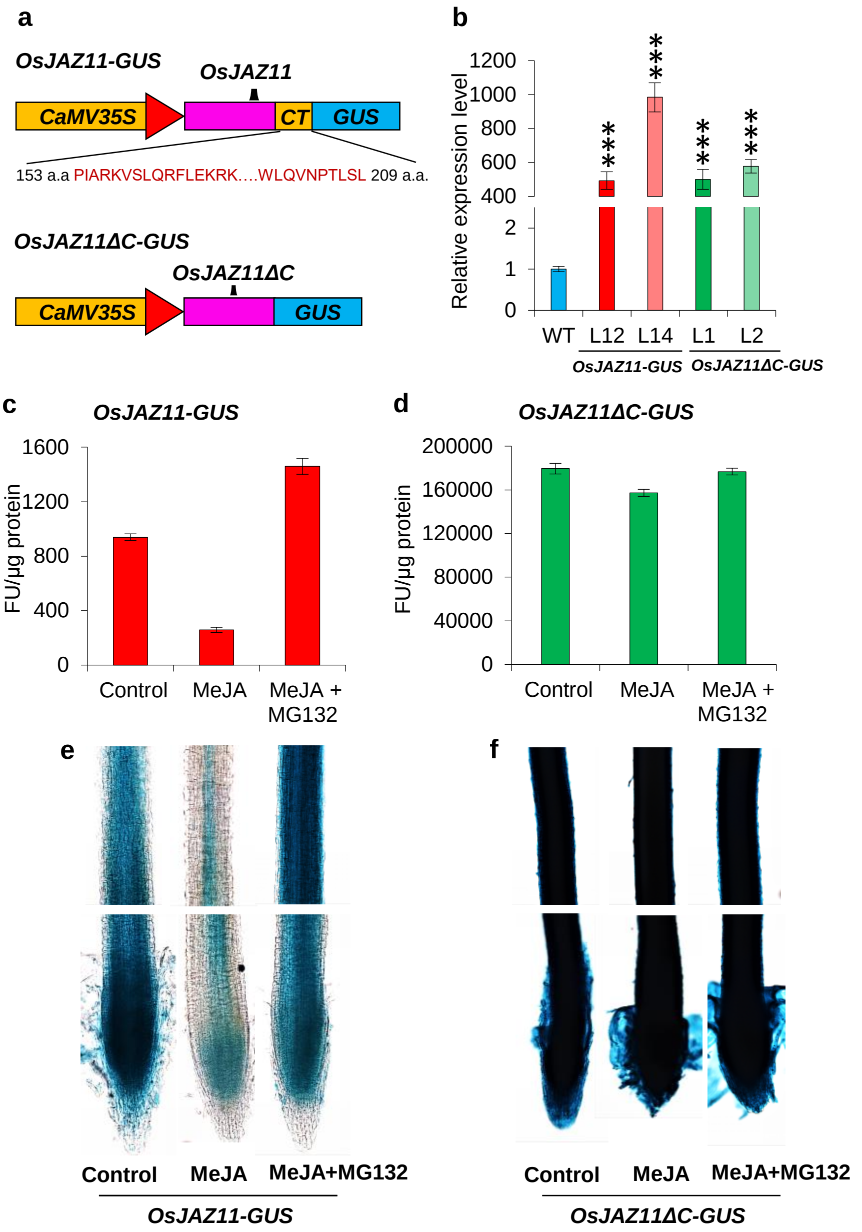

Supplementary Fig. S10 OsJAZ11 shows *in vivo* repressor activity. **a** Raising of *OsJAZ11* translational reporters. Schematic representation of *OsJAZ11-GUS* (with Jas motif) and *OsJAZ11ΔC-GUS* (without Jas motif) fusion constructs in pCAMBIA1301. In *OsJAZ11ΔC-GUS,* 57 a.a. (153- 209 a.a.) from C-terminal (CT) end of OsJAZ11 ORF was deleted. This deleted region also contains Jas motif. **b** Relative expression levels of *OsJAZ11* in WT, *OsJAZ11-GUS* (L12, L14) and *OsJAZ11ΔC-GUS* (L1, L2) transgenics. Expression levels were measured by RT-qPCR in roots of thirty-day-old plants. Each bar represents mean of three biological replicates with standard error. Significant differences between WT and transgenics were determined by Student's *t*-test. Asterisks; *** indicate *P* value, ≤ 0.001 (*n*=3). **c-d** Fluorometric quantitation of GUS signals in roots of fifteen-day-old *OsJAZ11-GUS* (L14) and *OsJAZ11ΔC-GUS* (L1) transgenics subjected to MeJA and/or MG132 treatments. Plants were treated with 100 µM MeJA with or without the proteasome inhibitor 100 µM MG132 for 1 h. DMSO treated seedlings were used as control. Each bar represents mean of four biological replicates with standard error (*n*=4). **e-f** Representative images showing GUS signals in root tips of fifteen-day-old *OsJAZ11-GUS* (L14) and *OsJAZ11ΔC-GUS* (L1) transgenics treated MeJA and/or MG132 treatments.

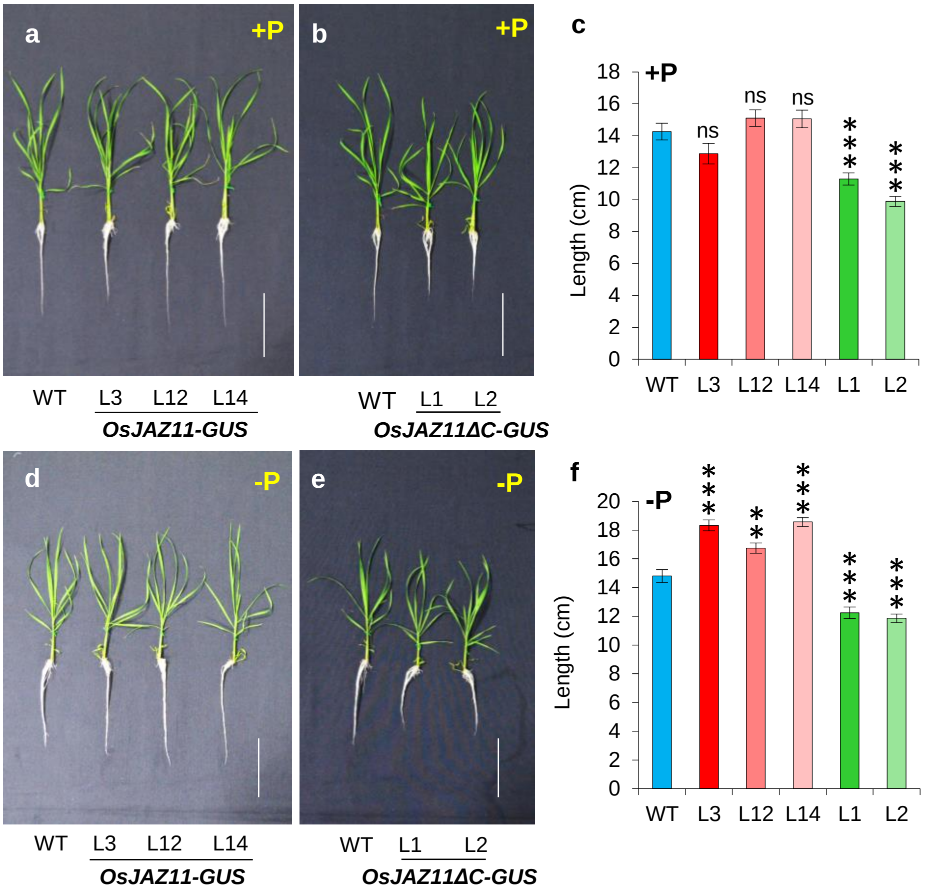

Supplementary Fig. S11 Root length of fifteen-day-old WT and *OsJAZ11* translational reporters. Root phenotype of fifteen-day-old *OsJAZ11-GUS* (**a**, **d**) and *OsJAZ11ΔC-GUS* lines (**b**, **e**) compared to WT under +P (**a**, **b**) and -P (**d**, **e**) conditions. For imaging three representative plants of each line were stacked together. Scale bar = 10 cm. **c** Quantitation of root lengths of WT and translational reporters under +P conditions. **f** Quantitation of root lengths of WT and translational reporters under -P conditions. Each bar represents mean of ten biological replicates with standard error. Significant differences between WT and transgenics were determined by Student's *t*-test. Asterisks; ** and *** indicate *P* values, ≤ 0.01 and 0.001, respectively. Ns denotes no significant differences between WT and transgenics (*n*=10).

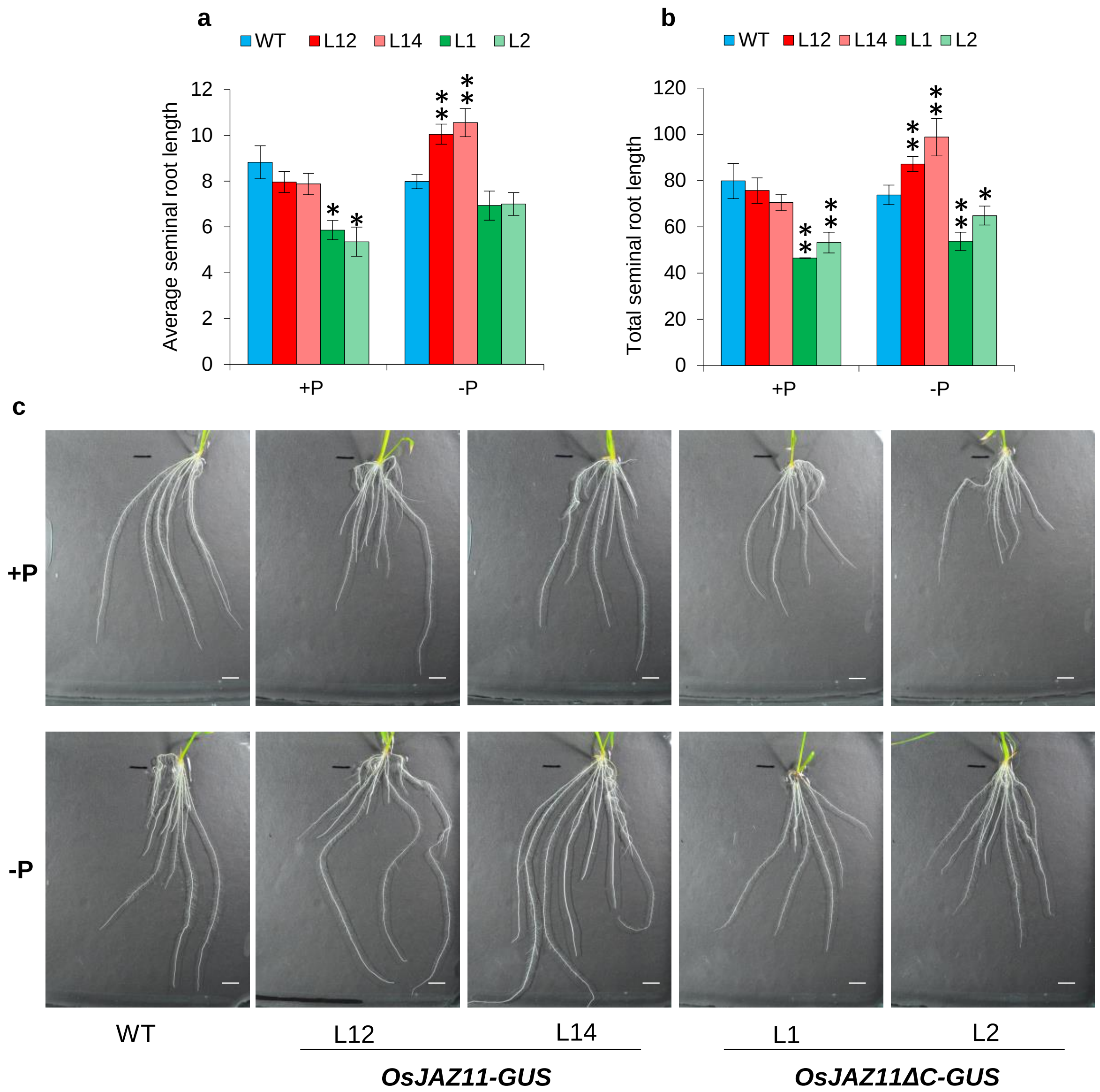

Supplementary Fig. S12 *OsJAZ11-GUS* lines developed longer seminal roots. Average seminal root length (**a**) and total seminal root length (**b**) of fifteen-day-old WT, *OsJAZ11-GUS* (L12, L14) and *OsJAZ11ΔC-GUS* (L1, L2) lines under +P and -P conditions. Each bar represents mean from four replicates with standard error. Significant differences between WT and transgenics were determined by Student's *t*-test. Asterisks; * and ** indicate *P* values, ≤ 0.05 and 0.01, respectively (*n*=4). **c** Representative images of fifteen-day-old WT and *OsJAZ11* translational reporters under +P and -P conditions. White line at bottom of each image denotes scale of 1 cm.

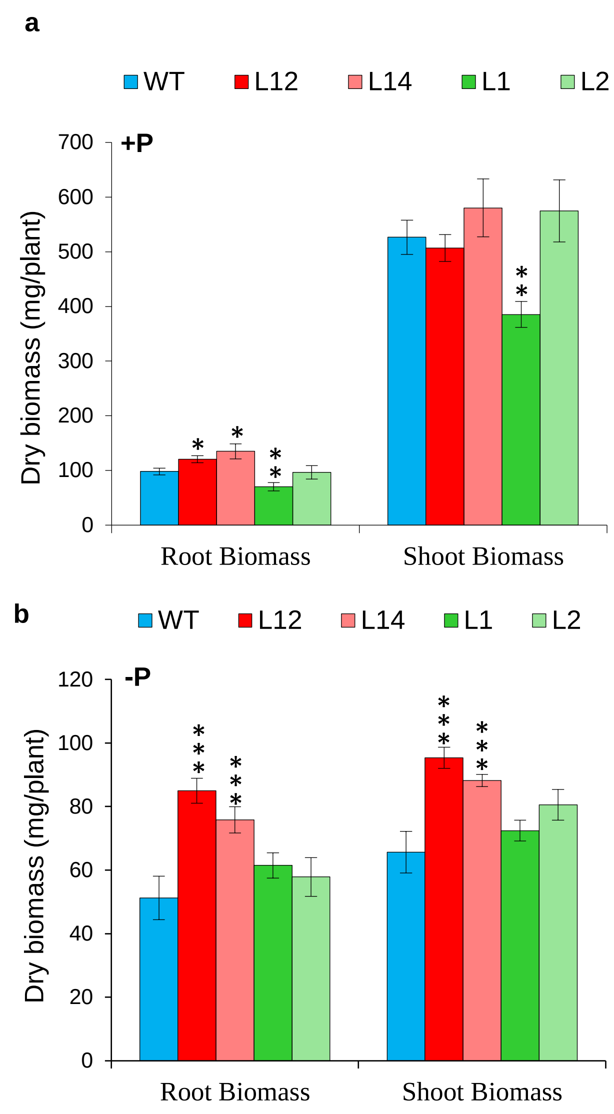

Supplementary Fig. S13 *OsJAZ11-GUS* lines accumulated more biomass under Pi deficiency. Root and shoot dry biomass of thirty-day-old WT, *OsJAZ11-GUS* (L12, L14) and *OsJAZ11ΔC-GUS* (L1, L2) lines under +P (**a**) and -P (**b**) conditions. Each bar represents mean of ten biological replicates with standard error. Significant differences between WT and transgenics were determined by Student's *t*-test. Asterisks; *, ** and *** indicate *P* values, ≤ 0.05, 0.01 and 0.001, respectively (*n*=10).

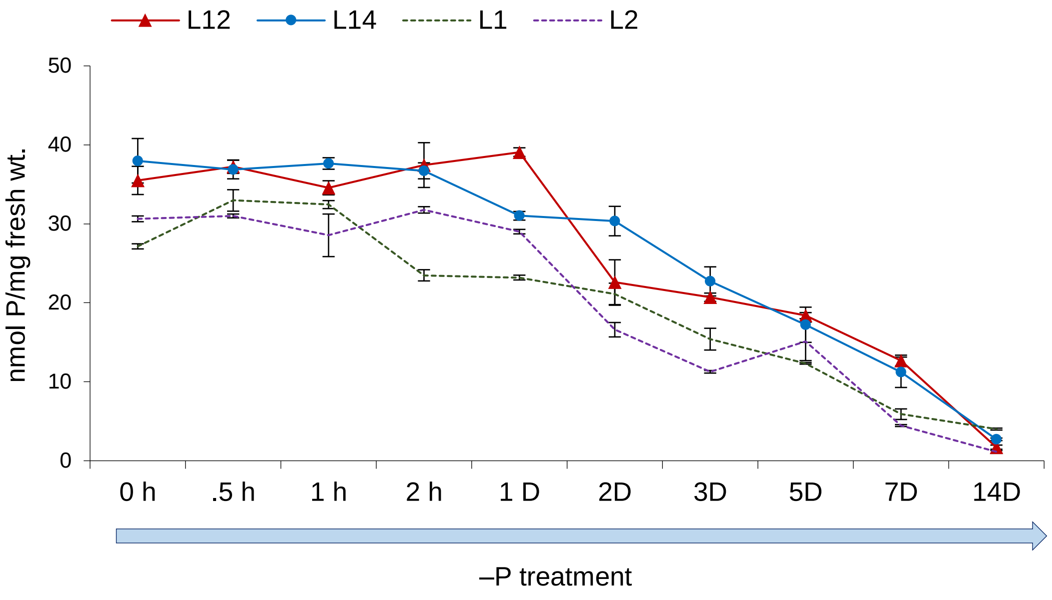

Supplementary Fig. S14 *OsJAZ11*-GUS transgenics accumulated higher Pi than *OsJAZ11ΔC-GUS* transgenics. Seven-day-old rice seedlings of *OsJAZ11*-GUS (L12, L14) and *OsJAZ11ΔC-GUS* (L1, L2) lines grown under +P conditions were transferred to –P conditions. Soluble Pi was measured at indicated time-points. Means were calculated from three biological replicates. Error bars indicate standard error (*n*=3).

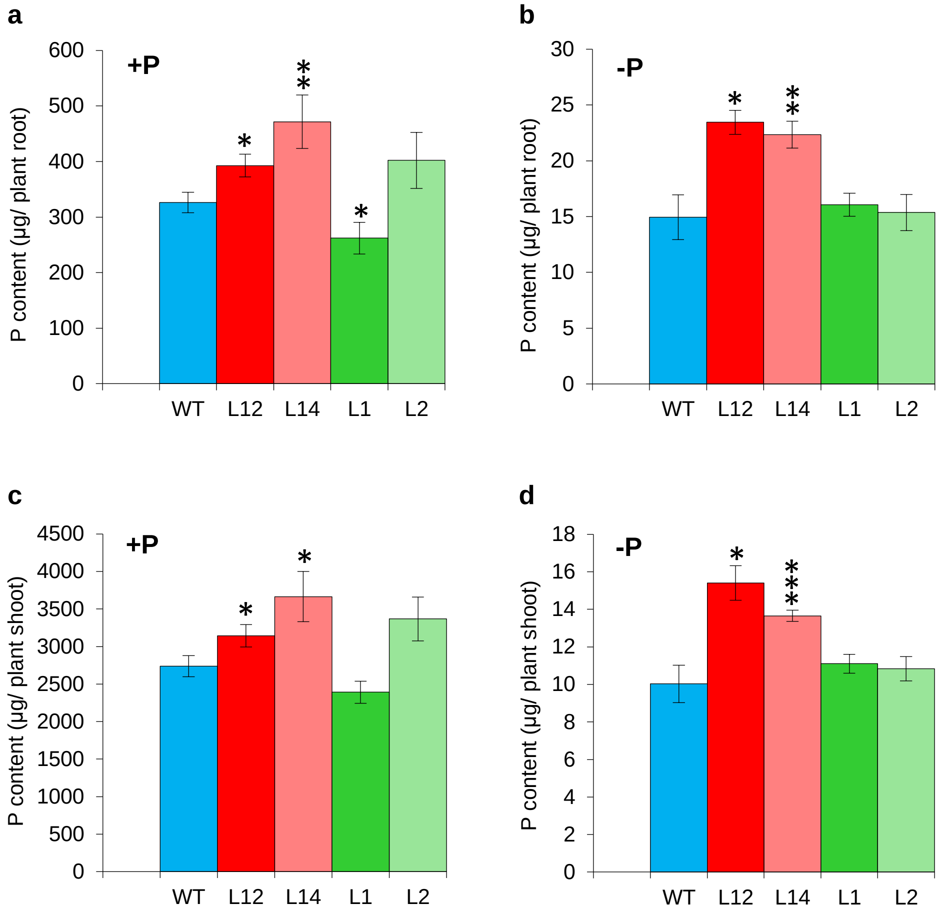

Supplementary Fig. S15 *OsJAZ11-GUS* lines showed higher phosphorous uptake under Pi deficiency. **a-b** P content of roots of thirty-day-old plants of WT, *OsJAZ11-GUS* (L12, L14) and *OsJAZ11ΔC-GUS* (L1, L2) lines under +P and -P conditions. **c-d** P content of shoots of thirty-day-old plants of WT, *OsJAZ11-GUS* (L12, L14) and *OsJAZ11ΔC-GUS* (L1, L2) lines under +P and -P conditions. Each bar displays means of ten biological replicates with standard error. Significant differences between WT and transgenics were determined by Student's *t*-test. Asterisks; *, ** and *** indicate *P* values, ≤ 0.05, 0.01 and 0.001, respectively (*n*=10).

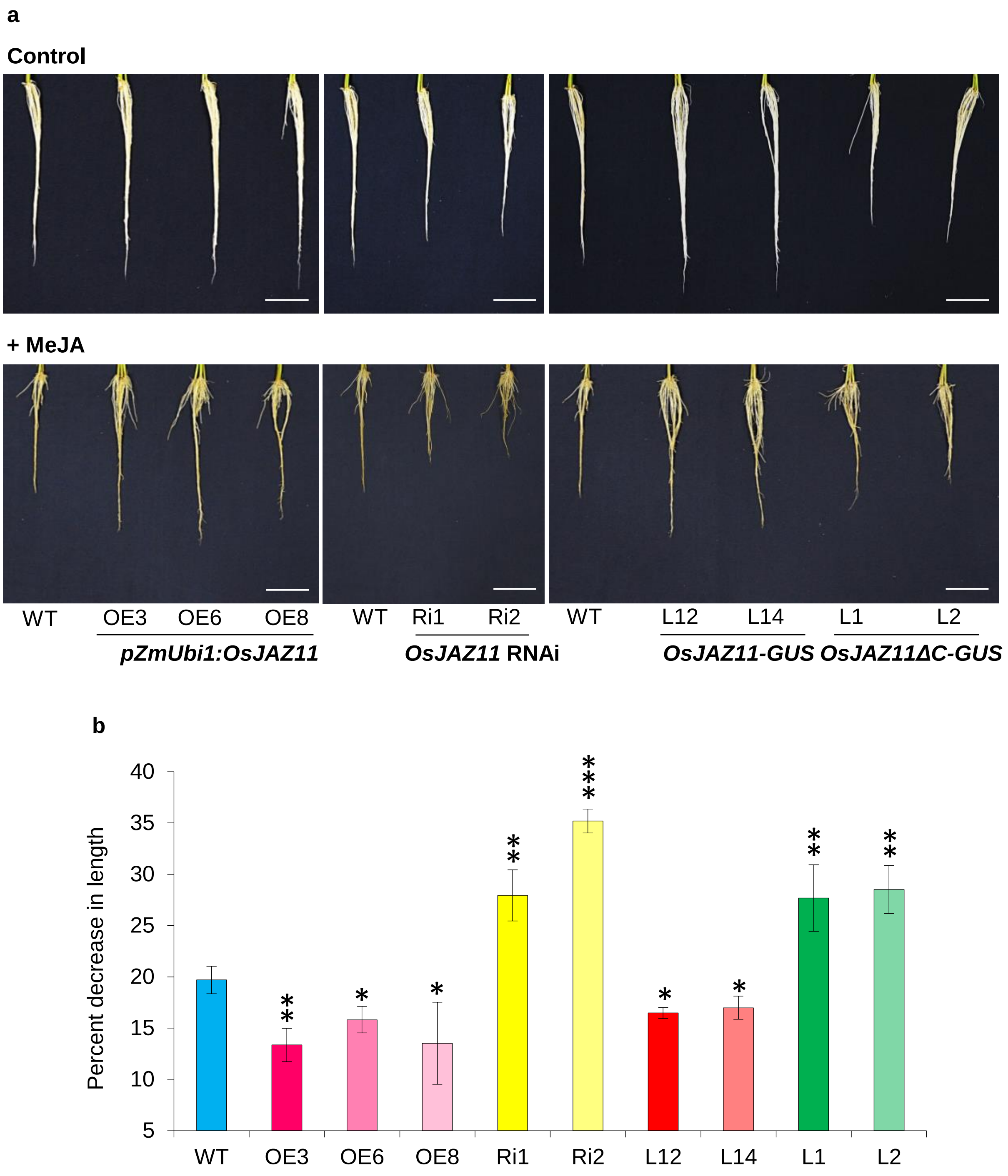

Supplementary Fig. S16 JA sensitivity assay of *OsJAZ11* transgenics. **a** Root images of WT and *OsJAZ11* transgenics subjected to DMSO (control) and MeJA (10 µM) treatment. Seven-day-old rice seedlings were treated with 10 µM MeJA or DMSO (control) for 14 days. For imaging three representative plants of each line were stacked together. Scale bar = 5 cm. **b** Percent reduction in root lengths of WT and *OsJAZ11* transgenics after MeJA treatment. Reduction in root length under MeJA treatment was compared to control conditions (*n*=10). Significant differences between WT and transgenics were determined by Student's *t*-test. Asterisks; *, ** and *** indicate *P* values, ≤ 0.05, 0.01 and 0.001, respectively (*n*=10).

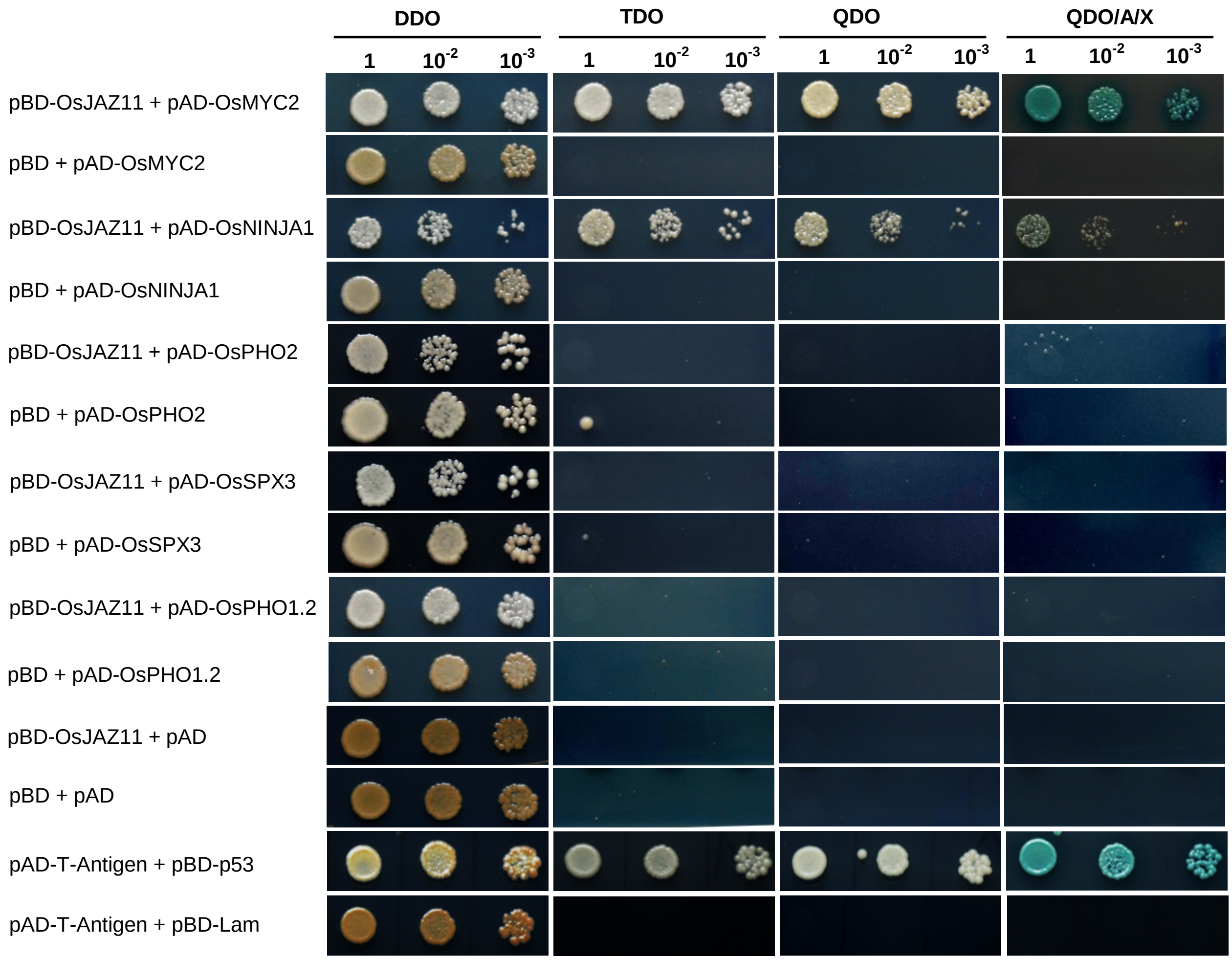

Supplementary Fig. S17 Interactions assays of OsJAZ11 with JA and Pi signaling proteins. Yeast two-hybrid interaction assays between bait plasmid, pBD-OsJAZ11 and prey plasmids, pAD-OsMYC2, pAD-OsNINJA1, pAD-OsPHO2, pAD-OsSPX3, pAD-OsPHO1.2. Serial dilutions of Y2H Gold strain co-transformed with AD (prey) and BD (bait) clones were spotted on DDO (SD-Leu/-Trp), TDO (SD- Leu/-Trp/-His), QDO (SD- Leu/-Trp/-His/-Ade) and QDO/A/X (QDO + Aureobasidin A + X-α-Gal). pBD and pAD indicates empty BD (pGBKT7) and AD (pGADT7) vectors, respectively. Interaction between pAD-T-Antigen and pBD-p53 was used as positive control whereas interaction between pAD-T-Antigen and pBD-Lam was used as a negative control.

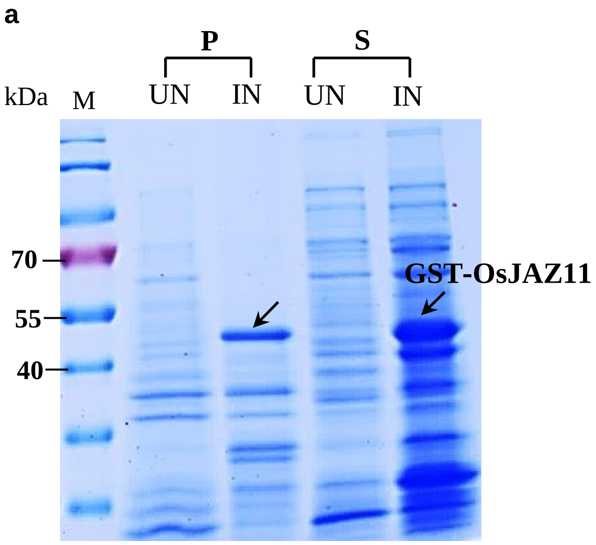

Supplementary Fig. S18 Induction and purification of OsJAZ11-GST. **a** Overexpression of OsJAZ11-GST in *E. coli* BL21(DE3) induced by IPTG. Induced (IN) and uninduced (UN) total protein fractions were resolved on 12% SDS-PAGE gel. Gel was stained with Coomassie blue. P and S implies proteins in pellet (insoluble) and supernatant (soluble) fractions. 20 µg of total protein was loaded in each lane. **b** Coomassie-stained SDS-PAGE gel showing 49.9 kDa purified recombinant OsJAZ11-GST protein.

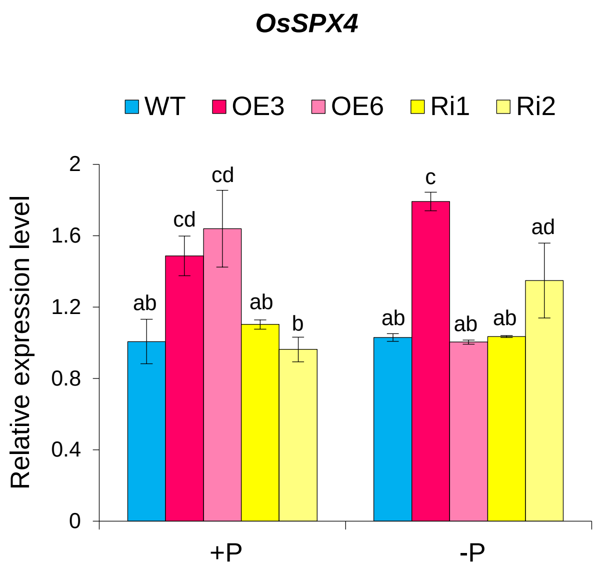

Supplementary Fig. S19 Expression of *OsSPX4* in WT and *OsJAZ11* transgenics. Relative expression levels of *OsSPX4* in roots of thirty-day-old WT and *OsJAZ11* transgenics under +P and -P conditions. Each bar is the mean of three independent replicates with standard error. Different letters on top of each bar denotes significant differences determined by one-way ANOVA followed by Duncan's multiple comparison test ($\alpha < 0.05$) (n=3).

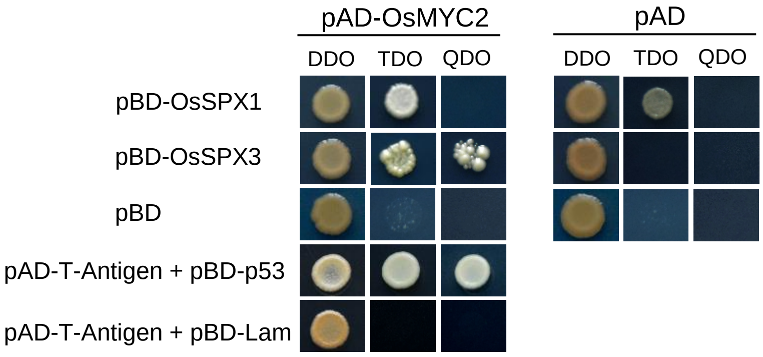

Supplementary Fig. S20 Interactions assays of OsMYC2 with SPX proteins. Yeast two-hybrid interaction assays between pAD-OsMYC2 and pBD-OsSPX (OsSPX1 and OsSPX3). Cells of yeast strain, Y2H Gold co-transformed with AD (prey) and BD (bait) clones were spotted on DDO (SD-Leu/-Trp), TDO (SD-Leu/-Trp/-His) and QDO (SD- Leu/-Trp/-His/-Ade). pBD and pAD indicates empty BD (pGBKT7) and AD (pGADT7) vectors, respectively. Interaction between pAD-T-Antigen and pBD-p53 was used as positive control whereas interaction between pAD-T-Antigen and pBD-Lam was used as a negative control.

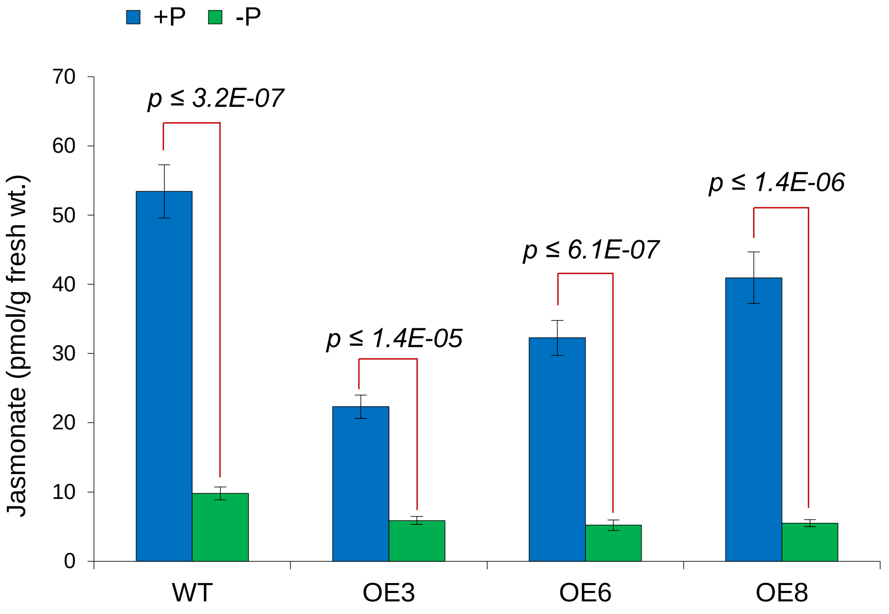

Supplementary Fig. S21 Pi deficiency reduces JA levels in rice. JA content in roots of thirty-day-old WT and *OsJAZ11* OE transgenics grown under +P and -P conditions. Each bar represents mean of six independent replicates with standard error. Significant differences between WT and transgenics were evaluated by Student's *t*-test (n=6).

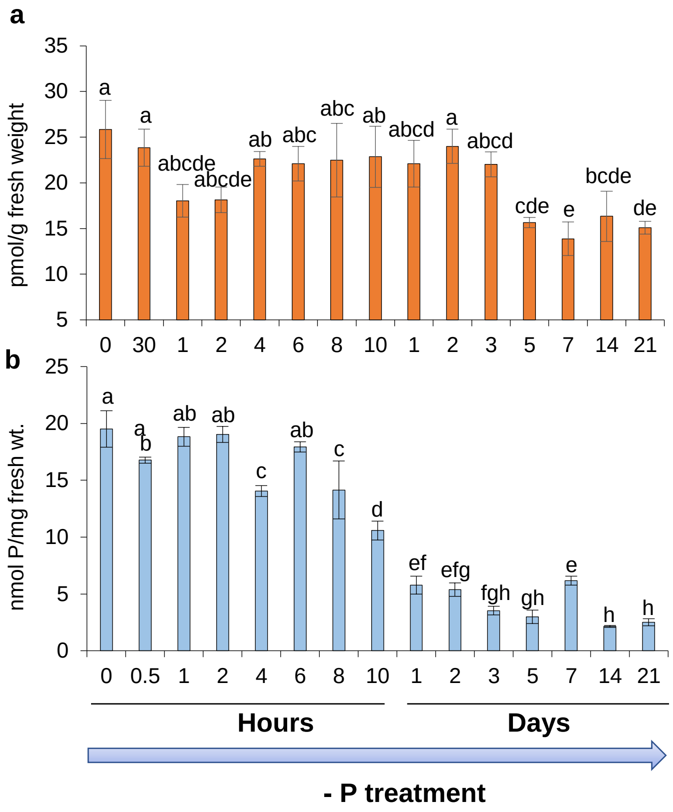

Supplementary Fig. S22 JA levels in rice under progressive Pi deficiency. JA levels (**a**) and soluble Pi content (**b**) in roots of PB1 seedlings. Seven-day-old rice seedlings grown under +P conditions were transferred to –P conditions. Soluble Pi and JA levels were measured at indicated time-points. For JA and Pi measurements means were calculated from six (n=6) and four (n=4) biological replicates, respectively. Error bars indicate standard error. Different letters on top of each bar denotes significant differences determined by one-way ANOVA followed by Duncan's multiple comparison test ($\alpha < 0.05$).

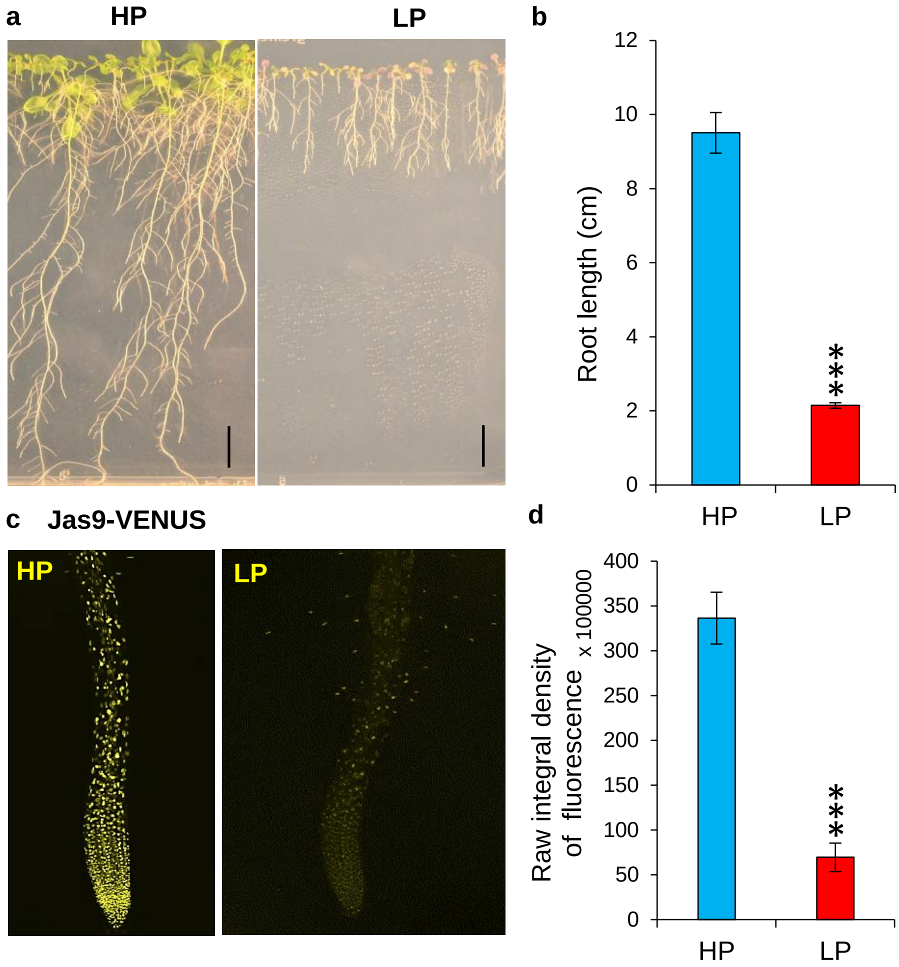

Supplementary Fig. S23 Pi deficiency leads to root length inhibition and increased JA levels in *Arabidopsis*. **a** Plant phenotype of ten-day-old *Arabidopsis* seedlings (Col-0) grown under Pi sufficient (HP) and deficient (LP) conditions Scale bar = 1 cm. **b** Quantitation of primary root length of *Arabidopsis* seedling grown under Pi sufficient (HP) and deficient (LP) conditions. Each bar represent mean of ten replicates with standard error. **c** Representative SP5 confocal images showing Jas9-VENUS fluorescence in primary root tips of *Arabidopsis* under HP and LP conditions. **d** Quantitation of VENUS fluorescence under HP and LP conditions. Significant differences between HP and LP conditions were determined by Student's *t*-test. Asterisks; *** indicate P value ≤ 0.001 (n=10).

Table S1. List of primers used in the study

	F (5'-3')	R (5'-3')
OsJAZ11_pANIC6B	CACCGAAGGTTTCGTCCTCGTT	CCATAGGTCCGACAGGCATCAA
OsJAZ11_pANIC8B	CACCAAGAAAGACGATGGC	TATAAGATCAGCATGCATGAAAG
OsJAZ11_pMDC163	CACCATCAATGCACCTAGGTGC	GTTGTAGCTCTAGCAAAATCAGCTAG
OsJAZ11_pCAMBIA1301	TGCTTAAGATCTAATGGCCGGTAGTAGCGA	TGCTTAAGATCTACCAGGCTGAGAGTGGGGT
OsJAZ11ΔC_pCAMBIA1301	TGCTTAAGATCTAATGGCCGGTAGTAGCGA	TGCTTAAGATCTACCATGTCTGGTAGGCAAGGC
OsJAZ11_pGEX4T1	TGCTTAGGATCCATGGCCGGTAGTAGCGAGC	GAGTTCGAATTCTCACAGGCTGAGAGTGGGGT
OsSPX1_pET28a	AATTGGATCCATGAAGTTTGGGAAGAGCCTGAGTAG	GATCGAATTCTCATTTGGCGGCCTGCTC
OsJAZ11_qRT_pCAMBIA	ACGCTGAAAGACACGCAAGA	TGGTCAGCTGCGCCTTCT
OsJAZ11_qRT_pANIC6B/pANIC8B	AATTCTTTCATGCATGCTGATCTTA	AGGTCCGACAGGCATCAATC
OsSPX1_qRT	TATATGAAGGGCACGGTCGC	ACAGTAGAGCTTCCGCTTCG
OsSPX2_qRT	CACCACCGACCTCCTCTACAA	GTTCGTTCGTCGGCATGAG
OsSPX3_qRT	GACGTACGGGCGGTTCTCG	GGATCGGATCGGATGGACTG
OsSPX4_qRT	GCCAGCCACGTGATGAATG	AGGGCCTCGGAGGATTAAAC
OsSPX5_qRT	ATGCCACATATTGCGACGAG	TCGATTTCAATTTGCTGCCAAAAG
OsPT1_qRT	CCTCGACATCGCCTTCTACAG	CACCCGACCTTGCTGAAGAT
OsPT4_qRT	TCGGCGTGCGCAACT	GGCACCAGGAACGTGAAGAA
OsPT8_qRT	GTCCCGGGCTACTGGTTCA	TGAAAAACCCTAGCAGCTGGAT
OsPT9_qRT	CCGGCTACATCGTGCTCTACT	AGGATGAAGGTGGTGGTGTTG
OsIPS1_qRT	AAGGGCAGGGCACACTCCACATTATC	ATTAGAGCAAGGACCGAAACACAAAC
OsGDPD5_qRT	TCCCCCAACCGATCTTGTC	GAAAGTGTATGGATGCACCTGAAG
OsMGDG3_qRT	AGTCGGGAATGTGCCTTACG	CTCGGGTCCTTGGAGAACAC
OsPAP3b_qRT	GCTCGATTCGCCTTCTACGA	TTTGCTGAAGCTCCAGTGGT
OsPAP10a_qRT	CGCCATCCTGGACATCAAG	TTGCGGTGCCACGTGTAG
OsJAZ4_qRT	GAGTGCCAATGACAACAAGTCATC	TGATTCGTCGCGGTTGCT
OsJAZ8_qRT	GAAGGCTCAACAGCTGACCAT	TTGGTGGACGGGAAGTTCTC
OsJAZ9_qRT	CGGTCGAGTTGGAAGATGGTT	GGTCAGGCTCGGCGAAAT
OSAOS1_qRT	CGCCTCGGCATTGCA	AGCGTCGGGAACAGGATCTT
OsAOS2_qRT	TGCCCATGATCATCGAGGAT	ACGCGTACAGCGCCTTGTA
OsOPR1_qRT	TCGCTGCTGGCGGGTAT	TAGCCATCAGCCACCACCTT
OsMYC2_qRT	AAATCCATTCCACCCCATACC	CCGTCCAAAGGTTCATCGAT
OsUBQ5_qRT	ACCACTTCGACCGCCACTACT	ACGCCTAAGCCTGCTGGTT
OsJAZ11_P1BS_EMSA	GAGTCACATCGGATATACATACACATATTT	AAATATGTGTATGTATATCCGATGTGACTC
OsJAZ11_mP1BS_EMSA	GAGTCACATCTGCGCGAAATACACATATTT	AAATATGTGTATTTCGCGCAGATGTGACTC
OsSPX2_PIBS_EMSA	TATCCCCGTCACGAATATTCCTCTTCCTCC	GGAGGAAGAGGAATATTCGTGACGGGGATA
OsJAZ11_BD	CACCATGGCCGGTAGTAGC	CAGGCTGAGAGTGGGGTTC

OsSPX1_AD/BD	CACCATGAAGTTTGGGAAGAGCCTG	TTTGGCGGCCTGCTCAATCAC
OsSPX3_AD/BD	CACCATGAAGTTTGGGAAGAG	GGCATAAAAAAACTGTAAACTTGGA
OsMYC2_AD	CACCATGTGGGTTTTGTTATCTCCTCTCCTCACCAC	CCGGGCGGCGGTGCCAG
OsPHO1;2_AD/BD	CACCATGGTGAAGTTCTCGAGG	ATCAGTTTCAAGTTCACGGAATGGTAAT
OsPHO2_AD	CACCATGGATCTATATGCAATTGACTCGGACACT	CGGGCTGCAGGGGATGCC
OsNINJA1_AD	AGAATTCCATATGGACGATGAGAATGGCCT	TGCTTAGAATTCTTAGTTTTGGGCTGAGGCTG

Table S2. List of interacting proteins identified in *OsJAZ11* OE lines using MS using Exactive™ Plus Orbitrap Mass Spectrometer

Accession	Description	Sum PEP Score	Coverage [%]	No. of Peptides	No. of AAs	MW [kDa]	pI	Biological Process	Molecular Function	Gene Symbol	KEGG Pathways
Q8GRS2	Protein TIFY 11c OS=Oryza sativa subsp. japonica OX=39947 GN=TIFY11C PE=1 SV=1	15.393	31	5	209	22	5.74	metabolic process; regulation of biological process; response to stimulus	protein binding	Os03g0180900; LOC4331833	Plant hormone signal transduction
Q0J8A4	Glyceraldehyde-3-phosphate dehydrogenase 1, cytosolic OS=Oryza sativa subsp. japonica OX=39947 GN=GAPC1 PE=1 SV=1	15.044	27	7	337	36.4	7.11	metabolic process	catalytic activity; nucleotide binding	Os08g0126300; LOC4344564	Biosynthesis of secondary metabolites; Metabolic pathways; Biosynthesis of amino acids; Carbon fixation in photosynthetic organisms; Glycolysis / Gluconeogenesis; Carbon metabolism
Q7FAH2	Glyceraldehyde-3-phosphate dehydrogenase 2, cytosolic OS=Oryza sativa subsp. japonica OX=39947 GN=GAPC2 PE=1 SV=1	10.571	17	5	337	36.8	6.81	metabolic process	catalytic activity; nucleotide binding	Os04g0486600; LOC4336216	Biosynthesis of secondary metabolites; Metabolic pathways; Biosynthesis of amino acids; Carbon fixation in photosynthetic organisms; Glycolysis / Gluconeogenesis; Carbon metabolism
Q2R8Z5	Alcohol dehydrogenase 1 OS=Oryza sativa subsp. japonica OX=39947 GN=ADH1 PE=2 SV=2	6.257	6	1	379	41	6.65	metabolic process	catalytic activity; metal ion binding	Os11g0210300; LOC4350053	Biosynthesis of secondary metabolites; Fatty acid degradation; Metabolic pathways; Glycolysis / Gluconeogenesis; Tyrosine metabolism; alpha-Linolenic acid metabolism
P93438	S-adenosylmethionine synthase 2 OS=Oryza sativa subsp. japonica OX=39947 GN=SAM2 PE=2 SV=1	5.578	15	3	394	42.9	6.05	metabolic process	catalytic activity; metal ion binding; nucleotide binding	OSJNBa0011P19.5; Os01g0323600; LOC4326996	Cysteine and methionine metabolism; Biosynthesis of secondary metabolites; Metabolic pathways; Biosynthesis of amino acids
Q2QS14	UDP-glucose 6-dehydrogenase 4 OS=Oryza sativa subsp. japonica OX=39947 GN=UGD4 PE=2 SV=1	4.755	9	3	480	52.8	6.13	metabolic process	catalytic activity; nucleotide binding	Os12g0443500; LOC4352146	Pentose and glucuronate interconversions; Metabolic pathways; Ascorbate and aldarate metabolism; Amino sugar and nucleotide sugar metabolism
Q9XGP7	Tricin synthase 1 OS=Oryza sativa subsp. japonica OX=39947 GN=ROMT-15 PE=1 SV=1	3.858	17	2	252	27.8	5.31	metabolic process	catalytic activity; metal ion binding	Os08g0498100; LOC4345934	
Q2QS13	UDP-glucose 6-dehydrogenase 5 OS=Oryza sativa subsp. japonica OX=39947 GN=UGD5 PE=2 SV=1	3.607	9	3	480	52.9	6.13	metabolic process	catalytic activity; nucleotide binding	Os12g0443600; LOC4352147	Pentose and glucuronate interconversions; Metabolic pathways; Ascorbate and

											aldarate metabolism; Amino sugar and nucleotide sugar metabolism
P31924	Sucrose synthase 1 OS=Oryza sativa subsp. japonica OX=39947 GN=SUS1 PE=1 SV=1	3.247	2	1	816	92.8	6.38	metabolic process; response to stimulus	catalytic activity	Os03g0401300; LOC4333062	Starch and sucrose metabolism; Metabolic pathways
Q9FR35	Peroxiredoxin-2C OS=Oryza sativa subsp. japonica OX=39947 GN=PRXIIC PE=1 SV=1	3.075	14	1	162	17.3	5.88	cellular homeostasis; metabolic process; regulation of biological process	antioxidant activity; catalytic activity	Os01g0675100; LOC4325511	
Q0J7U6	Protein TOPLESS-RELATED PROTEIN 2 OS=Oryza sativa subsp. japonica OX=39947 GN=TPR2 PE=1 SV=1	2.898	3	3	1133	124.7	7.28	metabolic process; regulation of biological process	protein binding	Os08g0162100; LOC4344728	
P51823	ADP-ribosylation factor 2 OS=Oryza sativa subsp. japonica OX=39947 GN=ARF PE=2 SV=2	2.698	25	3	181	20.7	6.95	metabolic process; regulation of biological process; response to stimulus; transport	catalytic activity; nucleotide binding; protein binding; signal transducer activity; transporter activity	Os05g0489600; LOC4339157	Endocytosis
A3C6D7	Actin-2 OS=Oryza sativa subsp. japonica OX=39947 GN=ACT2 PE=2 SV=1	2.319	8	2	377	41.7	5.49		nucleotide binding	Os10g0510000; LOC4349087	
P45960	Tubulin beta-4 chain OS=Oryza sativa subsp. japonica OX=39947 GN=TUBB4 PE=2 SV=1	1.976	5	2	447	50.3	4.86	cell organization and biogenesis; cellular component movement	catalytic activity; nucleotide binding; structural molecule activity	Os01g0805900; LOC4327550	
Q53M52	Tubulin alpha-2 chain OS=Oryza sativa subsp. japonica OX=39947 GN=TUBA PE=2 SV=1	1.432	4	1	451	49.7	4.96	cell organization and biogenesis	catalytic activity; nucleotide binding; structural molecule activity	Os11g0247300; LOC4350197	Phagosome
Q2QLY4	5-methyltetrahydropteroyltriglutamate--homocysteine methyltransferase 2 OS=Oryza sativa subsp. japonica OX=39947 GN=Os12g0624000 PE=2 SV=1	1.141	2	1	766	84.6	6.3	metabolic process	catalytic activity; metal ion binding	Os12g0624000; LOC4352833	Selenocompound metabolism; Cysteine and methionine metabolism; Biosynthesis of secondary metabolites; Metabolic pathways; Biosynthesis of amino acids
Q0J4P2	Heat shock protein 81-1 OS=Oryza sativa subsp. japonica OX=39947 GN=HSP81-1 PE=3 SV=2	1.019	4	1	699	80.1	5.07	metabolic process; response to stimulus	nucleotide binding; protein binding	OJ1118_A06.20; Os08g0500700; LOC4345951	Plant-pathogen interaction; Protein processing in endoplasmic reticulum
P93431	Ribulose bisphosphate carboxylase/oxygenase activase, chloroplastic OS=Oryza sativa subsp. japonica OX=39947 GN=RCA PE=1 SV=2	0.969	4	1	466	51.4	5.62	regulation of biological process	enzyme regulator activity; nucleotide binding	Os11g0707000; LOC4351224	

O64937	Elongation factor 1-alpha OS=Oryza sativa subsp. japonica OX=39947 GN=REFA1 PE=2 SV=2	0.823	2	1	447	49.3	9.06	metabolic process	catalytic activity; nucleotide binding; RNA binding	Os03g0177900; LOC4331812; Os03g0178000; LOC4331813; Os03g0177400; LOC4331810; Os03g0177500; LOC4331811	RNA transport
P14655	Glutamine synthetase, chloroplastic OS=Oryza sativa subsp. japonica OX=39947 GN=GLN2 PE=1 SV=1	0.799	4	1	428	46.6	6.34	metabolic process; response to stimulus	catalytic activity; nucleotide binding	Os04g0659100; LOC4337272	Arginine biosynthesis; Nitrogen metabolism; Metabolic pathways; Biosynthesis of amino acids; Alanine, aspartate and glutamate metabolism; Glyoxylate and dicarboxylate metabolism
Q10L71	Villin-2 OS=Oryza sativa subsp. japonica OX=39947 GN=VLN2 PE=2 SV=1	0.699	2	1	966	106.1	5.82	cell organization and biogenesis; regulation of biological process; response to stimulus	protein binding	Os03g0356700; LOC4332869	
P42211	Aspartic proteinase OS=Oryza sativa subsp. japonica OX=39947 GN=RAP PE=2 SV=2	0.631	5	1	496	54.1	6.15	metabolic process	catalytic activity	Os05g0137400; LOC4337744	
P51431	Ubiquitin-40S ribosomal protein S27a-2 OS=Oryza sativa subsp. japonica OX=39947 GN=RPS27AB PE=2 SV=3	0.612	10	1	155	17.7	9.8	metabolic process	metal ion binding; protein binding; structural molecule activity	Os05g0160200; LOC4337883	Ribosome
Q01859	ATP synthase subunit beta, mitochondrial OS=Oryza sativa subsp. japonica OX=39947 GN=ATPB PE=1 SV=2	0.585	7	1	552	58.9	6.37	metabolic process; transport	catalytic activity; nucleotide binding; transporter activity	Os05g0553000; LOC4339546	Oxidative phosphorylation; Metabolic pathways
Q9MAX5	Coatomer subunit zeta-1 OS=Oryza sativa subsp. japonica OX=39947 GN=COPZ1 PE=2 SV=1	0.571	11	1	177	19.4	4.73	transport		Os05g0461000; LOC4339020	
Q5VRW2	Transcription factor NIGTH1 OS=Oryza sativa subsp. japonica OX=39947 GN=NHO1 PE=1 SV=2	0.528	5	1	507	53	6.02	cell organization and biogenesis; metabolic process; regulation of biological process; response to stimulus	DNA binding; protein binding	Os01g0176700; LOC4326224	
Q10HL3	Endoribonuclease Dicer homolog 2a OS=Oryza sativa subsp. japonica OX=39947 GN=DCL2A PE=2 SV=1	0.5	1	1	1410	158.4	6.86	metabolic process; regulation of biological process; response to stimulus	catalytic activity; DNA binding; metal ion binding; nucleotide binding; protein binding; RNA binding	Os03g0583900; LOC4333337	
O82451	Probable glutathione S-transferase GSTF2 OS=Oryza sativa subsp.	0.5	9	1	215	24	6.13	metabolic process	catalytic activity; protein binding	Os01g0764000; LOC4326887	Glutathione metabolism

	japonica OX=39947 GN=GSTF2 PE=1 SV=3										
Q9AUV6	UDP-glucose 6-dehydrogenase 3 OS=Oryza sativa subsp. japonica OX=39947 GN=UGD3 PE=2 SV=1	3.607	9	3	480	52.9	6.13	metabolic process	catalytic activity; nucleotide binding	Os03g0757900; LOC4334169	Pentose and glucuronate interconversions; Metabolic pathways; Ascorbate and aldarate metabolism; Amino sugar and nucleotide sugar metabolism
Q10NY2	Protein TPR3 OS=Oryza sativa subsp. japonica OX=39947 GN=TPR3 PE=1 SV=1	2.898	3	3	1133	125.1	7.14	regulation of biological process	protein binding	Os03g0254700; LOC4332285	
Q06396	ADP-ribosylation factor 1 OS=Oryza sativa subsp. japonica OX=39947 GN=Os01g0813400 PE=2 SV=3	2.698	25	3	181	20.7	6.95	regulation of biological process; response to stimulus; transport	catalytic activity; nucleotide binding; protein binding; signal transducer activity; transporter activity	Os01g0813400; LOC4327591	Endocytosis
Q76FS2	Tubulin beta-8 chain OS=Oryza sativa subsp. japonica OX=39947 GN=TUBB8 PE=2 SV=1	1.976	5	2	446	49.6	4.89	cell organization and biogenesis	catalytic activity; nucleotide binding; structural molecule activity	Os03g0661300; LOC4333632	Phagosome
Q43594	Tubulin beta-1 chain OS=Oryza sativa subsp. japonica OX=39947 GN=TUBB1 PE=2 SV=2	1.976	5	2	447	50.3	4.83	cell organization and biogenesis	catalytic activity; nucleotide binding; structural molecule activity	Os01g0282800; LOC4326917	Phagosome
P46265	Tubulin beta-5 chain OS=Oryza sativa subsp. japonica OX=39947 GN=TUBB5 PE=1 SV=1	1.976	5	2	447	50.1	4.83	cell organization and biogenesis; cellular component movement	catalytic activity; nucleotide binding; structural molecule activity	Os02g0167300; LOC4328420	Phagosome
Q76FS3	Tubulin beta-6 chain OS=Oryza sativa subsp. japonica OX=39947 GN=TUBB6 PE=2 SV=1	1.976	5	2	444	49.9	4.91	cell organization and biogenesis	catalytic activity; nucleotide binding; structural molecule activity	Os05g0413200; LOC4338790	Phagosome
P37832	Tubulin beta-7 chain OS=Oryza sativa subsp. japonica OX=39947 GN=TUBB7 PE=2 SV=2	1.976	5	2	444	49.8	4.87	cell organization and biogenesis; cellular component movement	catalytic activity; nucleotide binding; structural molecule activity	Os03g0780600; LOC4334309	Phagosome
Q40665	Tubulin beta-3 chain OS=Oryza sativa subsp. japonica OX=39947 GN=TUBB3 PE=2 SV=2	1.976	5	2	446	50.1	4.83	cell organization and biogenesis	catalytic activity; nucleotide binding; structural molecule activity	Os06g0671900; LOC4341810	Phagosome
Q8H7U1	Tubulin beta-2 chain OS=Oryza sativa subsp. japonica OX=39947 GN=TUBB2 PE=2 SV=1	1.976	5	2	447	50.2	4.83	cell organization and biogenesis	catalytic activity; nucleotide binding; structural molecule activity	Os03g0105600; LOC4331315	Phagosome
P28752	Tubulin alpha-1 chain OS=Oryza sativa subsp. japonica OX=39947 GN=TUBA1 PE=1 SV=1	1.432	4	1	450	49.6	5.06	cell organization and biogenesis	catalytic activity; nucleotide binding;	Os07g0574800; LOC4343694	Phagosome

									structural molecule activity		
Q2QLY5	5-methyltetrahydropteroyltriglutamate--homocysteine methyltransferase 1 OS=Oryza sativa subsp. japonica OX=39947 GN=Os12g0623900 PE=2 SV=1	1.141	2	1	766	84.5	6.3	metabolic process	catalytic activity; metal ion binding	Os12g0623900; LOC4352832	Selenocompound metabolism; Cysteine and methionine metabolism; Biosynthesis of secondary metabolites; Metabolic pathways; Biosynthesis of amino acids
P0CH34	Ubiquitin-60S ribosomal protein L40-1 OS=Oryza sativa subsp. japonica OX=39947 GN=Ub-CEP52-1 PE=2 SV=1	0.612	12	1	129	14.8	9.92	metabolic process	protein binding; structural molecule activity	Os03g0234200; LOC4332169; Os09g0568400; LOC4347896	Ribosome
P0C031	Ubiquitin-NEDD8-like protein RUB2 OS=Oryza sativa subsp. japonica OX=39947 GN=RUB2 PE=2 SV=2	0.612	10	1	153	17.1	6.06		protein binding	Os06g0650100; LOC4341684	
Q9ARZ9	Ubiquitin-40S ribosomal protein S27a-1 OS=Oryza sativa subsp. japonica OX=39947 GN=RPS27AA PE=2 SV=1	0.612	10	1	155	17.7	9.77	metabolic process	metal ion binding; protein binding; structural molecule activity	Os01g0328400; LOC4326977	Ribosome
P0C030	Ubiquitin-NEDD8-like protein RUB1 OS=Oryza sativa subsp. japonica OX=39947 GN=RUB1 PE=2 SV=2	0.612	10	1	153	17.1	6.06		protein binding	Os09g0420800; LOC4347085	
Q58G87	Polyubiquitin 3 OS=Oryza sativa subsp. japonica OX=39947 GN=UBQ3 PE=2 SV=2	0.612	17	1	381	42.7	7.58	response to stimulus	protein binding	OSJNBa0089N06.4; Os04g0628100; LOC4337080	
Q69LX2	Endoribonuclease Dicer homolog 2b OS=Oryza sativa subsp. japonica OX=39947 GN=DCL2B PE=2 SV=2	0.5	1	1	1377	154.7	7.15	metabolic process; regulation of biological process; response to stimulus	catalytic activity; DNA binding; metal ion binding; nucleotide binding; protein binding; RNA binding	Os09g0315100; LOC4346696	
Q6K5G8	Glyceraldehyde-3-phosphate dehydrogenase 3, cytosolic OS=Oryza sativa subsp. japonica OX=39947 GN=GAPC3 PE=2 SV=1	6.657	9	3	337	36.5	7.88	metabolic process	catalytic activity; nucleotide binding	Os02g0601300; LOC4329892	Biosynthesis of secondary metabolites; Metabolic pathways; Biosynthesis of amino acids; Carbon fixation in photosynthetic organisms; Glycolysis / Gluconeogenesis; Carbon metabolism
Q0DKY4	S-adenosylmethionine synthase 1 OS=Oryza sativa subsp. japonica OX=39947 GN=SAM1 PE=2 SV=1	4.233	12	2	396	43.2	6.14	metabolic process	catalytic activity; metal ion binding; nucleotide binding	Os05g0135700; LOC4337733	Cysteine and methionine metabolism; Biosynthesis of secondary metabolites; Metabolic pathways; Biosynthesis of amino acids
Q9LGU6	S-adenosylmethionine synthase 3 OS=Oryza sativa subsp. japonica OX=39947 GN=METK3 PE=2 SV=1	4.233	12	2	396	43.3	5.41	metabolic process	catalytic activity; metal ion binding; nucleotide binding	Os01g0293000; LOC4325163	Cysteine and methionine metabolism; Biosynthesis of secondary metabolites;

											Metabolic pathways; Biosynthesis of amino acids
Q5NBT9	Protein TPR1 OS=Oryza sativa subsp. japonica OX=39947 GN=TPR1 PE=1 SV=1	2.042	2	2	1129	125	7.18	regulation of biological process	protein binding	Os01g0254100; LOC4327709	
Q75GS4	UDP-glucose 6-dehydrogenase 1 OS=Oryza sativa subsp. japonica OX=39947 GN=UGD1 PE=3 SV=1	1.223	3	1	481	52.3	6.04	metabolic process	catalytic activity; nucleotide binding	Os03g0425600; LOC4333156	Pentose and glucuronate interconversions; Metabolic pathways; Ascorbate and aldarate metabolism; Amino sugar and nucleotide sugar metabolism
B7F958	UDP-glucose 6-dehydrogenase 2 OS=Oryza sativa subsp. japonica OX=39947 GN=UGD2 PE=2 SV=1	1.223	3	1	482	53.1	7.18	metabolic process	catalytic activity; nucleotide binding	Os03g0604200; LOC4333410	Pentose and glucuronate interconversions; Metabolic pathways; Ascorbate and aldarate metabolism; Amino sugar and nucleotide sugar metabolism
P0C540	Actin-7 OS=Oryza sativa subsp. japonica OX=39947 GN=ACT7 PE=3 SV=1	0.84	4	1	376	41.6	5.39		nucleotide binding	Os01g0866100; LOC4325068	
Q10DV7	Actin-1 OS=Oryza sativa subsp. japonica OX=39947 GN=ACT1 PE=2 SV=1	0.84	4	1	377	41.8	5.49		nucleotide binding	Os03g0718100; LOC4333919	
Q10AZ4	Actin-3 OS=Oryza sativa subsp. japonica OX=39947 GN=ACT3 PE=2 SV=1	0.84	4	1	377	41.7	5.49		nucleotide binding	Os03g0836000; LOC4334702	
